# Contrasting Gene Expression Profiles of Monocytes and Lymphocytes From Peste-Des-Petits-Ruminants Virus Infected Goats

**DOI:** 10.3389/fimmu.2019.01463

**Published:** 2019-07-05

**Authors:** Sajad Ahmad Wani, Amit Ranjan Sahu, Raja Ishaq Nabi Khan, Aruna Pandey, Shikha Saxena, Neelima Hosamani, Waseem Akram Malla, Dheeraj Chaudhary, Sonam Kanchan, Vaishali Sah, Kaushal Kishor Rajak, D. Muthuchelvan, Bina Mishra, Ashok Kumar Tiwari, Aditya P. Sahoo, Basavaraj Sajjanar, Yash Pal Singh, Ravi Kumar Gandham, Bishnu Prasad Mishra, Raj Kumar Singh

**Affiliations:** ^1^Division of Veterinary Biotechnology, ICAR-Indian Veterinary Research Institute (IVRI), Bareilly, India; ^2^Division of Pharmaceutics and Pharmaceutical Chemistry, The Ohio State University, Columbus, OH, United States; ^3^Genomics and Computational Biology, DBT-National Institute of Animal Biotechnology, Hyderabad, India; ^4^Division of Virology, ICAR-Indian Veterinary Research Institute (IVRI), Mukteswar, India; ^5^Division of Animal Genetics and Breeding, ICAR-Indian Veterinary Research Institute (IVRI), Bareilly, India; ^6^Division of Biological Products, ICAR-Indian Veterinary Research Institute, Bareilly, India; ^7^Division of Biological Standardization, ICAR-Indian Veterinary Research Institute, Bareilly, India; ^8^ICAR- Directorate of Foot and Mouth Disease, Mukteswar, India; ^9^ARIS Cell, ICAR-Indian Veterinary Research Institute (IVRI), Bareilly, India

**Keywords:** lymphocytes, monocytes, RNA sequencing, PPRV, goat, host-pathogen interaction

## Abstract

In this study, transcriptome analysis of PPRV infected PBMC subsets—T helper cells, T cytotoxic cells, monocytes, and B lymphocytes was done to delineate their role in host response. PPRV was found to infect lymphocytes and not monocytes. The established receptor for PPRV—SLAM was found downregulated in lymphocytes and non-differentially expressed in monocytes. A profound deviation in the global gene expression profile with a large number of unique upregulated genes (851) and downregulated genes (605) was observed in monocytes in comparison to lymphocytes. ISGs—ISG15, Mx1, Mx2, RSAD2, IFIT3, and IFIT5 that play a role in antiviral response and the genes for viral sensors—MDA5, LGP2, and RIG1, were found to be upregulated in lymphocytes and downregulated in monocytes. The transcription factors—IRF-7 and STAT-1 that regulate expression of most of the ISGs were found activated in lymphocytes and not in monocytes. Interferon signaling pathway and RIG1 like receptor signaling pathway were found activated in lymphocytes and not in monocytes. This contrast in gene expression profiles and signaling pathways indicated the predominant role of lymphocytes in generating the antiviral response against PPRV in goats, thus, giving us new insights into host response to PPRV.

## Introduction

*Peste des petits ruminants* (PPR) is an acute, highly contagious viral disease with high mortality (90%) and morbidity (100%) in sheep and goats and is classified as an OIE listed disease due to its economic relevance and severity (1). It is characterized by high fever, mucous membrane erosions, discharge through eyes and nose, enteritis and pneumonia, culminating in a fatal outcome (2). The causative agent is single-stranded, negative-sense RNA virus of genus *Morbillivirus* and family *Paramyxoviridae*. An investigation into the molecular determinants of the pathogenesis of PPRV is of considerable interest to understand the host-virus interaction and to devise strategies for its eradication.

PPR virus (PPRV) is a lymphotropic and epitheliotropic cell-associated virus, which uses peripheral blood mononuclear cell (PBMCs) as a vehicle for its dissemination within the host (3, 4). SLAM/CD150 established as a receptor for PPRV, is found on activated T cells, B cells, thymocytes and dendritic cells (5). Morbilliviruses infect both T and B lymphocytes (6, 7). PBMCs play a significant role in pathogen recognition and initiation of early innate immune response. This makes them a standard model for studying host-pathogen interactions in PPRV and other related infections (8–11). We have previously identified various immune modulating transcription factors and predicted an immune signaling pathway in response to Sungri/96 live attenuated PPRV vaccine strain *in vitro* in PBMCs. Our group has also deciphered miRNAome of lung and spleen of PPRV infected sheep and goats, identified and validated suitable reference genes for expression studies in PPRV infected tissues and revealed the role of miRNAome of PBMCs in regulating immune processes in PPRV infected goat (12–15).

The host immune response is often complicated due to differences in how different cell types receive a signal from different classes of receptors and produce distinct effector molecules (16). The individual role of PBMC subsets in terms of their contribution to immune response against Zaire ebolavirus and inactivated seasonal influenza virus infection has been explored through RNA-Sequencing analysis (17, 18). However, RNA-Sequencing of PPRV infected PBMC subsets has not been explored to date. Transcriptome analysis of the subpopulation of highly enriched circulating leukocytes and analysis of cell-specific pathways will help in understanding leukocyte regulatory networks in PPRV infection. In this study, subsets of peripheral blood leukocytes were isolated from control (0 day) and PPRV infected (9 dpi) goats through MACS technology and subjected to RNA-Sequencing. Subsequently, transcriptome analysis combined with system-level network analyses revealed the contrast between monocytes and lymphocytes with respect to expression of genes, signaling pathways and putative upstream regulators.

## Materials and Methods

### Animal Experiment, Ethics Statement, and Viral Infection

Highly virulent PPRV (Izatnagar/94, accession number KR140086.1) that is being maintained at National Morbillivirus Referral Laboratory, Indian Veterinary Research Institute, Muktehswar by animal-to-animal passages (19, 20) was used in the present study. The permission to conduct the study was granted by Indian Veterinary Research Institute Animal Ethics Committee (IVRI-IAEC) under the Committee for the Purpose of Control and Supervision of Experiments on Animals (CPCSEA), India, vide letter no 387/CPSCEA. Healthy goats (*n* = 4) that were negative for PPRV antibody by competitive-ELISA (21) and by serum neutralization test (SNT) (22), and for PPRV antigen by s-ELISA, were inoculated with the virus as mentioned in our earlier study (23). s-ELISA for detection of virus in nasal, ocular, buccal, and rectal swabs, PPRV N gene expression by qRT-PCR in PBMC subsets, histopathology, and immunohistochemistry of tissues were used for confirmation of infection.

### Isolation of T Helper Cells, T Cytotoxic Cells, B Lymphocytes, and Monocytes

Blood was collected from goats (*n* = 4) in heparin-coated vacutainer vials. PBMCs were isolated by using Ficol histopaque gradient method. PBMCs were strained through cell strainer of 0.40 micron. The PBMCs subsets were enriched by positive selection using indirect MACS technology (Milteny Biotech). Initially, the cell-specific surface marker FITC-conjugated primary antibodies, anti CD4^+^ (T helper cells, #MCA2213F), anti CD8^+^ (T cytotoxic cells, #MCA2216F), anti CD14^+^ (Monocytes, #MCA1568F), and anti CD21^+^ (B lymphocytes, #MCA1195F), were used. Subsequently, the cells were magnetically labeled with anti—FITC MicroBeads. Then the cell suspension was loaded on a miniMACS® column which was placed in the magnetic field of a MACS Separator. The magnetically labeled cells were retained in the column while the unlabeled cells run through. After removal of the column from the magnetic field, the magnetically retained cells were eluted as positively selected cell fraction. Cell sorting was done as per the manufacturer's protocol. Primary FITC-conjugated antibodies were titrated to determine the optimal dilution. Cells were kept on the ice and cold buffers were employed to minimize alterations in gene expression during labeling and sorting. The purity of the cells was further checked by flow cytometer. The cells were stored in RNA later for further use at −80°C.

### RNA-Sequencing of the Samples

The RNA-sequencing of each subset of PBMCs was carried out following the standard procedure as described in our previous study (20). Briefly, total RNA was isolated using the RNeasy Mini kit (Qiagen GmbH, Germany). Following the quality and integrity assessment on Bioanalyzer (Agilent Technologies, Inc), RNA (100 ng) was used for library preparation with the help of NEBNext Ultra RNA Library Prep Kit for Illumina (NewEngland Biolabs Inc.). The quality of the libraries was checked on Bioanalyzer and quantity was measured using a Qubit 2.0 Fluorometer (Life Technologies) and by qPCR. Library (1.3 ml, 1.8 pM) was denatured, diluted, and loaded onto a flow cell for sequencing. FastQC (Babraham Bioinformatics) was used for quality assessment of raw sequence data and prinseq-lite software (24) was used to remove reads of low quality (mean phred score 25) and short length (<50) for downstream analysis.

### Identification of Differentially Expressed Genes (DEGs)

**Figure 1:**
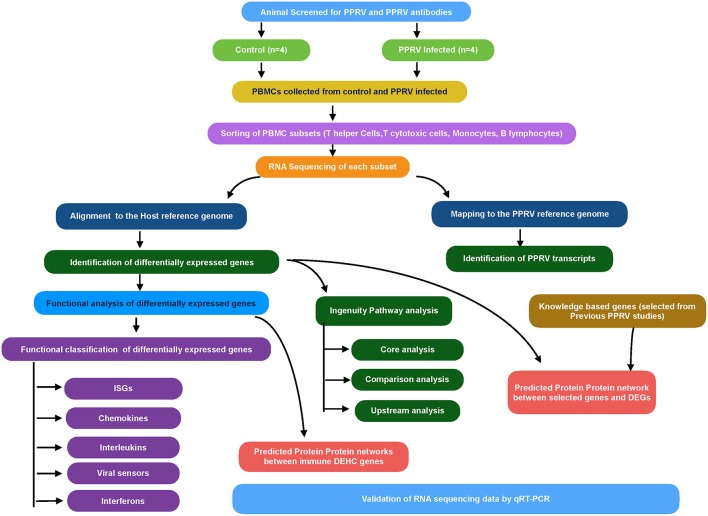
Workflow for RNA sequencing data analysis.

The overview of the transcriptome analysis is given in Figure 1. Initially, the quality-filtered reads from control and infected samples (0 days and 9 dpi) were mapped to *Capra hircus* genome using Bowtie2 (25). The mapped reads were then assembled using RNA-Seq by expectation maximization (RSEM) (26). The counts were used for calculating DEGs by use of R packages—EBSeq, DESeq2 and edgeR. The common DEGs from the three packages were used for downstream analysis while as fold changes for the corresponding genes was taken from DESeq2.

### Functional Analysis of DEGs

Using g:Profiler, the significantly expressed (*p* ≤ 0.05) DEGs were functionally annotated (27). The retrieved viral processes and immune system processes were chosen to find the genes associated with these processes. Further, the genes involved in immune-related functions were manually classified into antiviral interferon-stimulated genes (ISGs), chemokines, interleukins, viral sensors (PRRs), Interferons and Granzyme B, based on literature evidence and the type of associated immune process, to get comprehensive insights. Besides, 106 genes (= knowledge-based genes) were selected based on their role in host response against viruses from the earlier host-PPR virus interaction studies (8, 12) to explore variation in their expression across the PBMC subsets.

### Predicted Protein-Protein Interaction(PPI) Network

The Biological General Repository for Interaction Datasets (BioGRID) is a repository of protein-protein and genetic interactions for many species. Based on the interactions available in the BioGRID database, PPI network among the selected genes is retrieved (28). Initially, the DEGs obtained from transcriptome analysis were narrowed down to differentially expressed highly connected (DEHC) genes based on immune-related functions (from g:Profiler), fold change ≥±1.5 (Up or downregulated) and degree (calculated using igraph package) ≥5 for T helper cells, T cytotoxic cells, and B lymphocytes and degree ≥10 for monocytes. The PPI networks were constructed between the DEHC-DEHC genes, and between the knowledge-based genes and DEGs. The BioGRID database contains well-defined protein-protein interactions of human than in the case of *Bos taurus*. Considering protein interactions are conserved across species (29), orthologs in human were queried using g: Orth in g:profiler (27). The interactions involving the DEGs were extracted using customized perl scripts and were visualized in Cytoscape 3.3.0 (30).

### Identification of Viral Transcripts

The unmapped reads from all samples (control and infected) were mapped to PPRV reference genome (GenBank: AJ849636.2) (31) through Bowtie 2.0 for identification of viral transcripts.

### Ingenuity Pathway Analysis (IPA) Analysis

QIAGEN's IPA (QIAGEN, Redwood City, USA) was used to analyze the data of T helper cells, T cytotoxic cells, B lymphocytes, and monocytes. IPA has got its own database, Ingenuity Pathways Knowledge Base (IKB) that along with the list of DEGs were used to identify the canonical pathways and the most significant biological processes. Core analysis for each dataset was performed to know activated (Z score > 2) or inactivated (Z score < −2) canonical pathways. Also, upstream regulators (transcription factors, cytokines, and other molecules) were identified.

### Validation of DEGs by Quantitative Real-Time PCR (qRT-PCR)

To validate the expression of some selected genes, qRT-PCR was performed on Applied Biosystems 7500 Fast system. *GAPDH* was taken as the internal control as it was found to be the best suitable endogenous control in earlier studies in PPRV (12). The probe ID of selected genes used in the study for validation is given in Supplementary Table 1. Each of the samples was run in triplicates and relative expression of each gene was calculated using the 2^−ΔΔ*CT*^ method with control as the calibrator (32). Student's *t*-test was done in JMP9 (SAS Institute Inc., Cary, USA) to test the significance of difference. Differences between groups were considered significant at *P* ≤ 0.05.

## Results

To uncover the transcriptional variation underlying the host response to PPRV at PBMCs subtype level, enriched T helper cells, T cytotoxic cells, B lymphocytes, and monocytes were obtained by using MACS technology. The purity was found to be >90% through flow cytometry (data not shown). Then, we performed the global transcriptome profiling of T helper cells, T cytotoxic cells, and B lymphocytes and monocytes at 0 day (control) and 9 dpi.

### Viral Quantification by RNA-Sequencing

**Figure 2:**
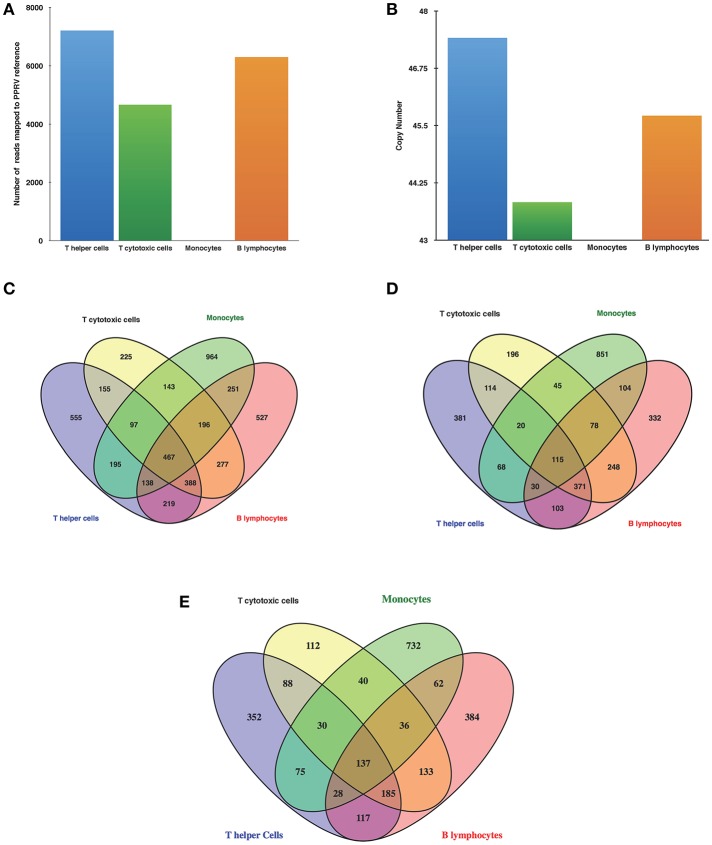
PPR virulent virus tropism in PBMCs subsets **(A)** Detection of viral reads through RNA Seq **(B)** N gene expression by q-RT-PCR of infected goats at 9 dpi. Venn diagrams representing unique/common **(C)** DEGs among cells **(D)** Upregulated genes among cells **(E)** Downregulated genes among cells.

Following alignment with Bowtie 2, several reads in the infected T helper cells, T cytotoxic cells, and B lymphocytes mapped to PPRV reference genome but no reads from infected monocyte mapped to PPRV genome (Figure 2A). PPRV N gene expression as confirmed by qRT-PCR was found in infected T helper cells, T cytotoxic cells, and B lymphocytes, but not in infected monocytes (Figure 2B).

### Identification of DEGs—Transcriptome Analysis of Isolated Cell Subpopulations Divulges Unique Gene Expression Patterns

The number of DEGs in T helper cells were 2,214 (1,202 upregulated, 1,012 downregulated), T cytotoxic cells−1,948 (1,187 upregulated, 761 downregulated), monocytes−2,451 (1,311 upregulated, 1,140 downregulated), and B lymphocytes−2,463 (1,381 upregulated, 1,082 downregulated). The number of upregulated genes was larger than the numbers of downregulated genes in all the cells. Furthermore, Venn diagrams were generated to examine the overlapping mRNA profiles between cells. This revealed 467 genes being common all subsets and 555, 225, 964, and 527 genes being unique in T helper cells, T cytotoxic cells, monocytes, and B lymphocytes, respectively (Figure 2C). Upon comparison of upregulated and downregulated genes 115 and 137 genes were common, respectively. The number of unique upregulated genes were 381, 196, 851, and 332 in T helper cells, T cytotoxic cells, monocytes, and B lymphocytes, respectively (Figure 2D) whereas number of unique downregulated genes were 352, 112, 732, and 384 in T helper cells, T cytotoxic cells, monocytes, and B lymphocytes, respectively (Figure 2E). Based on fold change values, top 20 DEGs showing marked upregulation and downregulation were enlisted in Supplementary Tables 2, 3, respectively. The list of top 20 upregulated and downregulated genes in monocytes were found to be unique with no overlap across other subsets.

### Transcriptome Landscape of DEGs Like Antiviral Interferon-Stimulated Genes (ISGs), Chemokines, Interleukins, Viral Sensors (PRRs), Signaling Lymphocytic Activation Molecule (SLAM), Interferons, Granzyme B

ISGs highly expressed in lymphocytes were, *ISG15, ISG20, IRF7, IFIT1, IFIT3, IFIT5, MX1, MX2, RELA, RSAD2, DDIT4, EIF2AK2, IFI6, OAS1X, PML*, and *RTP4*. Monocytes showed a contrasting ISGs profile with most of the genes downregulated (Supplementary Figure 1). Among the chemokines and their receptors, *CXCL10* was upregulated in lymphocytes and not differentially expressed in monocytes and *CXCR4* was upregulated in lymphocytes as well as monocytes (Supplementary Figure 2A). More number of interleukins and their receptors were differentially expressed in lymphocytes than in monocytes. *IL-1*β was upregulated in lymphocytes and not differentially expressed in monocytes. *IL-18* and *receptor IL1RN* were topmost upregulated interleukins in lymphocytes and monocytes, respectively (Supplementary Figure 2B). Among Viral RNA sensors, *DDX58* and *IFIH1* were found to be upregulated in lymphocytes and downregulated in monocytes. The differential expression of *DDX58* across subsets in comparison to TLR/NLR expression indicated the predominant role of RIG-I-like receptors under PPRV infection (Supplementary Figure 3). IFN α and IFN β were not differentially expressed in lymphocytes and monocytes. However, IFN receptors *IFNAR1* was downregulated in lymphocytes and monocytes while IFN Gamma receptors *IFNGR1* and *IFNGR2* were upregulated B lymphocytes (Supplementary Figure 3). SLAM was found to be downregulated in T helper cells (Log_2_ fold change −2.130), T cytotoxic cells (Log_2_ fold change −1.185), and B lymphocytes (Log_2_ fold change −2.204). On the contrary, it was not found to be differentially expressed in monocytes. Further, granzyme B (GZB) was found to be non-differentially expressed in lymphocytes and monocytes.

### DEGs Involved in Pathways in the Viral Process, Viral Life Cycle, Viral Genome Replication etc.

Among the DEGs, several genes were found to be involved in viral process, viral life cycle, viral genome replication, regulation of viral process, regulation of viral life cycle, regulation of viral genome replication, viral gene expression, and defense response to virus. The number of genes involved in above-mentioned processes was more in lymphocytes than in monocytes. Among these genes *ISG15, ISG20, IFIT3, MX2, IFIT1, IFIT2, IFIT5,OAS1X, MX1, DHX58, DDX58, HSPB1, DDIT4, RSAD2, EIF2AK2, NPC2, C19ORF66, ADAR*, and *DDX3X* were found to be upregulated in lymphocytes and downregulated in monocytes. Whereas, as *CXCL10, HMOX1, TMPRSS2, TNF, ICAM1, C1QBP, LEF1, EIF3D*, and *SNW1* were found to be upregulated in lymphocytes and not differentially expressed in monocytes (Supplementary Figure 4).

### DEGs Selected Through a Knowledge-Based Approach

Out of these 106 knowledge-based genes, 71, 72, 72, and 80 were differentially expressed in T helper cells, T cytotoxic cells, monocytes, and B lymphocytes, respectively. Most of the genes were found to be downregulated in monocytes but upregulated in lymphocytes. The innate antiviral genes—ISGs; *ISG15, ISG20, DDX58, DHX58, IRF3, IRF7, IFIT1, IFIT3, IFIT5, MX1, MX2*, and *RSAD2* were downregulated in monocytes indicating a dampened antiviral response of monocytes (Supplementary Figure 5).

### Predicted PPI Network of DEGs

DEGs regulating the immune processes were selected through functional analysis. The number of immune-related genes with cut off fold change ≥±1.5 and degree ≥5 were 269, 250, 70, 356 in T helper cells, T cytotoxic cells, monocytes, and B lymphocytes, respectively, and were designated as differentially expressed highly connected (DEHC) genes. As the number of interactions was higher in monocytes, the cut off was increased to degree ≥10 reducing the number of DEHC genes to 194. The PPI networks were constructed between immune DEHC genes; and between the genes selected through knowledge-based approach (106 genes) and DEGs, which revealed common hubs (Supplementary Figures 6–9). In the PPI networks, hubs explain the functional and structural importance of a network. The genes, which act as hubs in PPI networks in different cells are mentioned in the Supplementary Tables 4, 5. *ISG15, PML, MYC*, and *MAP3K5* genes were identified as common hubs in PPI network of immune DEHC-DEHC genes and *ISG15, STAT1*, and *BCL6* genes were identified as common hubs in PPI networks of selected genes and DEGs across all the subsets. The network hub genes (except *MAP3K5*) were downregulated in monocytes and upregulated in lymphocytes.

### Pathway Analysis by IPA

#### Core Analysis of T Helper Cells, T Cytotoxic Cells, B Lymphocytes, and Monocytes

Core analysis for each dataset was performed to know activated (Z score > 2) or inactivated (Z score < −2) canonical pathways. Moreover, the focus was on the apoptosis, cellular immune response, humoral immune response, cytokine signaling, and pathogen influenced signaling ingenuity canonical pathways.

##### T helper cells

**Figure 3:**
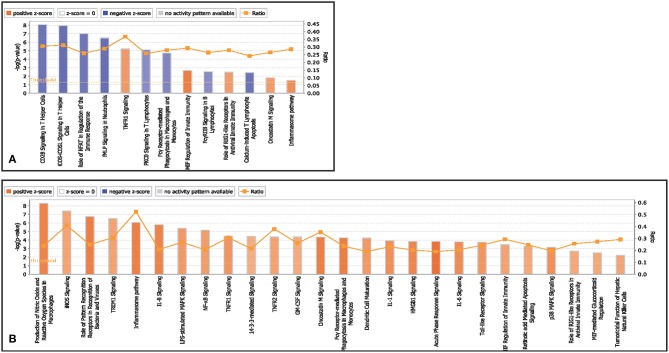
Canonical pathways activated/inactivated in **(A)** T helper cells **(B)** T cytotoxic cells of infected goats at 9 dpi generated in core analysis of Ingenuity pathway analysis tool. Orange color pathways are activated (>2) and blue color pathways are inactivated (<2). Height of the bar graphs indicates -log (*p*-value) and line graph showing the ratio of list genes found in each pathway over the total number of genes in that pathway.

Canonical pathways associated with T helper cells in infected goats at 9 dpi are represented in Figure 3A. The canonical pathway TNFR1 signaling was found to have the highest ratio of genes involved vis-a-vis the genes in the database. The top activated pathways based on Z score were TNFR1 signaling, MIF regulation of innate immunity and the role of RIG1 like receptors in antiviral immunity. The pathways—CD28 signaling in T Helper Cells, iCOS-iCOSL signaling in T helper cells, calcium-induced T lymphocyte apoptosis, the role of NFAT in the regulation of the immune response, Fc gamma receptor-mediated phagocytosis in macrophages and monocytes and PKC theta signaling in T Lymphocytes were found to be inactivated.

##### T cytotoxic cells

Canonical pathways associated with T cytotoxic cells in infected goats at 9 dpi are represented in Figure 3B. The top activated pathways based on Z score were production of NO and ROS in macrophages, iNOs signaling, role of PRRs in recognition of pathogens, inflammasome pathway, IL-8 pathway, Oncostatin M signaling, and acute phase response signaling. The canonical pathway inflammasome pathway TNFR1 signaling was found to have the highest ratio of genes involved vis-a-vis the genes in the database.

##### Monocytes

**Figure 4:**
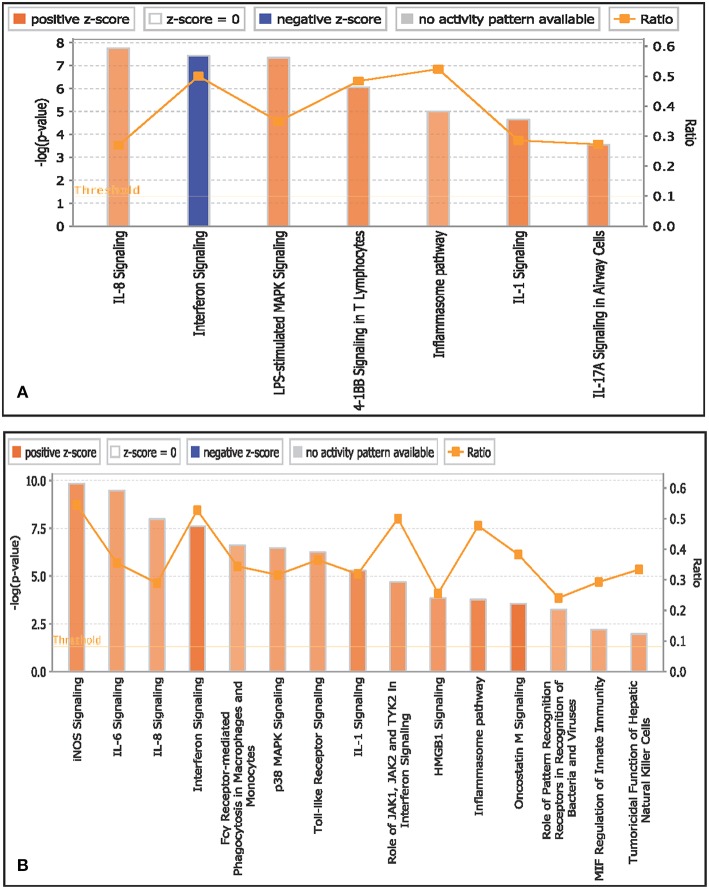
Canonical pathways activated/inactivated in **(A)** monocytes and **(B)** B lymphocytes of infected goats at 9 dpi generated in core analysis of Ingenuity pathway analysis tool. Orange color pathways are activated (>2) and blue color pathways are inactivated (<2). Height of the bar graphs indicates -log (*p*-value) and line graph show the ratio of list genes found in each pathway over the total number of genes in that pathway.

Canonical pathways associated with monocytes in infected goats at 9 dpi are represented in Figure 4A. The top activated pathways based on Z score were IL-8 signaling, LPS stimulated MAPK signaling, 41B signaling in T lymphocytes, and IL-1 signaling. Interferon signaling was the only pathway to be inactivated. The canonical pathway inflammasome pathway was found to have the highest ratio of genes involved vis-a-vis the genes in the database.

##### B lymphocytes

Canonical pathways associated with B lymphocytes in infected goats at 9 dpi are represented in Figure 4B. The top activated pathways based on Z score were interferon signaling, oncostatin M signaling, IL-1 signaling, inflammasome pathway, IL-1 signaling, acute phase response signaling, iNOS signaling, IL-6 signaling, p38 MAPK signaling, Toll-like receptor signaling, IL-8 signaling, MIF-mediated glucocorticoid regulation, and MIF regulation of innate immunity. The canonical pathways iNOS signaling and interferon signaling were found to have the highest ratio of genes involved vis-a-vis the genes in the database.

#### Comparison Analysis of Canonical Pathways Among T Helper Cells, T Cytotoxic Cells, B Lymphocytes, and Monocytes

Comparison of canonical pathways in T helper cells, T cytotoxic cells, monocytes, and B lymphocytes at 9 dpi in goats is represented in Supplementary Figure 10. Inflammasome pathway was activated in all cells. T cytotoxic cells had more number of activated pathways. MIF regulation of innate immunity, acute phase response signaling, acute phase response signaling, and Oncostatin M signaling were the topmost activated pathways in T helper cells, T cytotoxic cells, monocytes, and B lymphocytes, respectively.

**Figure 5:**
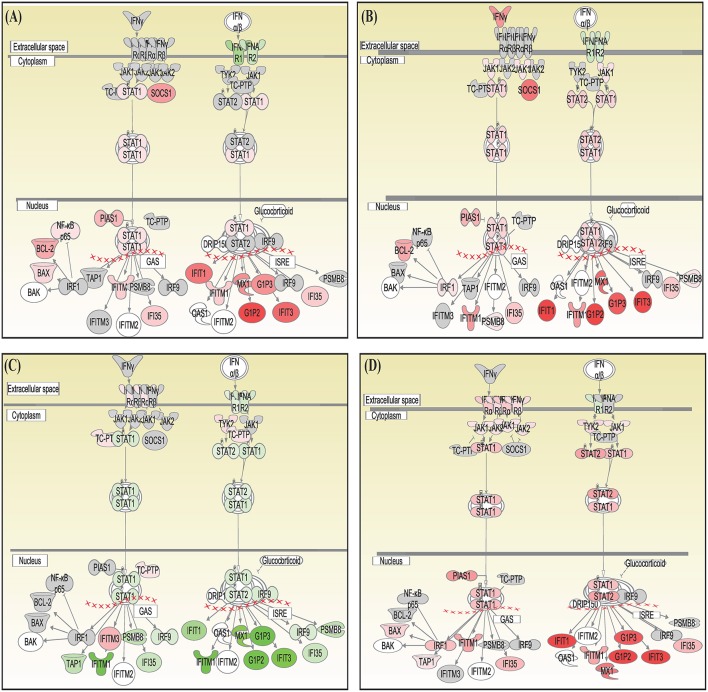
Canonical pathways generated in Ingenuity Pathway Analysis of interferon signaling pathway of DEGs in **(A)** T helper cells, **(B)** T cytotoxic cells, **(C)** monocytes, and **(D)** B lymphocytes of infected goats at 9 dpi. Genes that were upregulated are shown in red and downregulated in green. The intensity of red and green corresponds to an increase and decrease, respectively, in Log2 fold change. Genes in gray were not significantly differentially expressed and those in white are not present in the dataset but have been incorporated in the network through the relationship with other molecules by IPA. Symbol shape indicates gene function.

**Figure 6:**
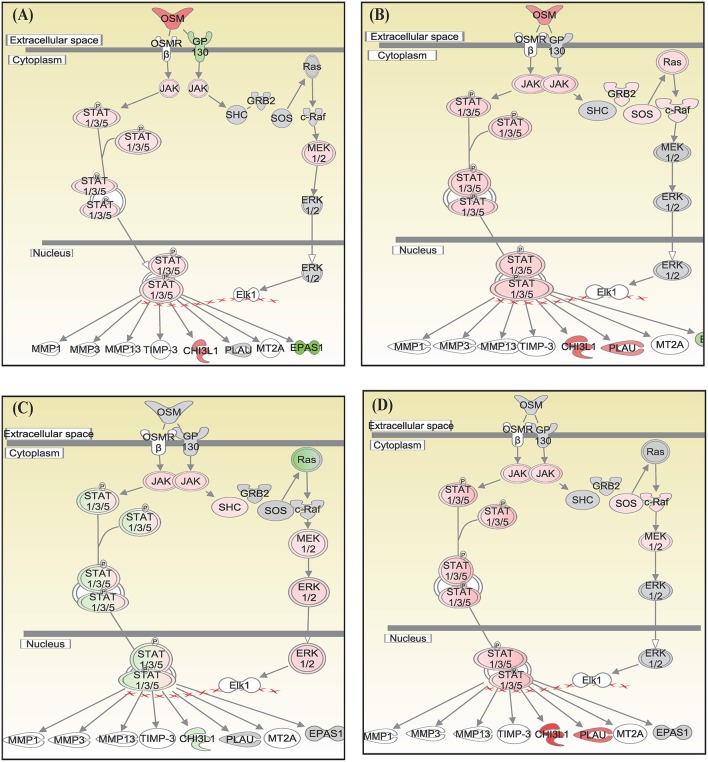
Canonical pathways generated in Ingenuity Pathway Analysis of Oncostatin M signaling pathway of DEGs in **(A)** T helper cells, **(B)** T cytotoxic cells, **(C)** monocytes, and **(D)** B lymphocytes of infected goats at 9 dpi. Genes that were upregulated are shown in red and downregulated in green. The intensity of red and green corresponds to an increase and decrease, respectively, in Log2 fold change. Genes in gray were not significantly differentially expressed and those in white are not present in the dataset but have been incorporated in the network through the relationship with other molecules by IPA. Symbol shape indicates gene function.

**Figure 7:**
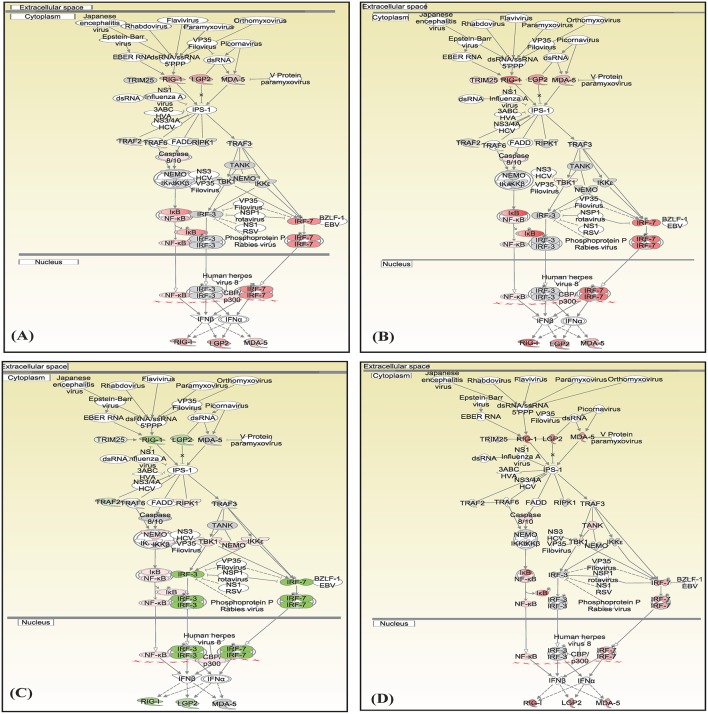
Canonical pathways generated in Ingenuity Pathway Analysis of the role of RIG 1 like receptors in antiviral immunity pathway of DEGs in **(A)** T helper cells, **(B)** T cytotoxic cells, **(C)** monocytes, and **(D)** B lymphocytes of infected goats at 9 dpi. Genes that were upregulated are shown in red and downregulated in green. The intensity of red and green corresponds to an increase and decrease, respectively, in Log2 fold change. Genes in gray were not significantly differentially expressed and those in white are not present in the dataset but have been incorporated in the network through the relationship with other molecules by IPA. Symbol shape indicates gene function.

**Figure 8:**
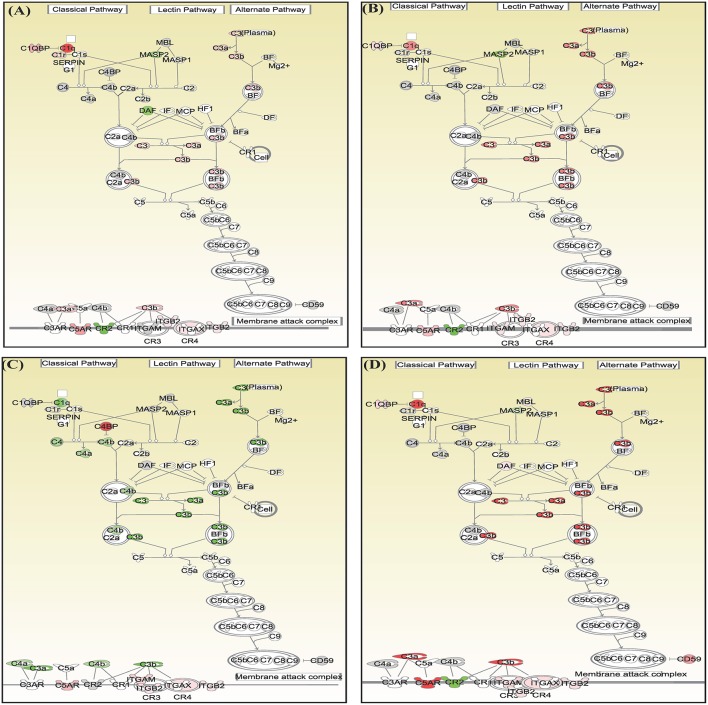
Canonical pathways generated in Ingenuity Pathway Analysis of role of complement system pathway of DEGs in **(A)** T helper cells, **(B)** T cytotoxic cells, **(C)** monocytes, and **(D)** B lymphocytes of infected goats at 9 dpi. Genes that were upregulated are shown in red and downregulated in green. The intensity of red and green corresponds to an increase and decrease, respectively, in Log2 fold change. Genes in gray were not significantly differentially expressed and those in white are not present in the dataset but have been incorporated in the network through the relationship with other molecules by IPA. Symbol shape indicates gene function.

Canonical pathways interferon signaling, Oncostatin M signaling, role of RIG 1 like receptors in antiviral immunity and complement system showed contrasting gene expression profiles in monocytes in comparison to lymphocytes. Interferon Signaling was found to be inactivated in monocytes (Z score −2.66) in comparison to T helper cells (Z score 0.77), T cytotoxic cells (Z score 1.88), and B lymphocytes (Z score 3.44). Genes in the interferon signaling network *GIP2* (*ISG15*), *IFIT1, IFIT3, MX1, IFI6, IFITM1, IFI35, STAT1*, and *STAT2* were significantly downregulated in monocytes. However, all these genes were upregulated in T helper cells, T cytotoxic cells, and B lymphocytes with varying degree of expression (Figure 5). Oncostatin M Signaling was found to be activated in T helper cells, T cytotoxic cells, and B lymphocytes. *OSM* was not differentially expressed and *STAT1* and *CH13L1* were downregulated in monocytes in contrast to their upregulation in T helper cells, T cytotoxic cells, and B lymphocytes (Figure 6). Role of RIG1-like Receptors in antiviral innate immunity was having lowest z score in monocytes (−0.83) in comparison to T helper cells (2.12), T cytotoxic cells (2.12), and B lymphocytes (1.89). A contrast in the expression of the genes involved in this pathway was also found in monocytes and lymphocytes (Figure 7). Complement system was found to be having negative z score in monocytes (−1.88) and a positive score in T helper cells (1.66), T cytotoxic cells (1.13), and B lymphocytes (0.37). Genes involved in the complement—*C1q, C3b, C3a*, and *C3* were upregulated in lymphocytes but downregulated in monocytes. *C1QBP* was upregulated in lymphocytes but not significantly expressed in monocytes (Figure 8).

#### Upstream Regulators, That Regulate DEGs in T Helper Cells, T Cytotoxic Cells, B Lymphocytes, and Monocytes

The number of TFs (activated/inactivated) differentially expressed and governing the DEGs in T helper cells, T cytotoxic cells, monocytes, and B lymphocytes were 21, 24, 10, and 30, respectively. The top four upregulated TFs in T helper cells were *KLF4, IRF7, XBP1*, and *MYC*; in T cytotoxic cells were *KLF4, ETS2, NFKB1A*, and *IRF7*; in monocytes were *EGR1, PPP1R13L*, and *STAT3* and in B Lymphocytes were *MXD1, KLF4, NFKBIA*, and *BCL6* (Supplementary Figure 11).

Master regulator, *IRF7*—transcription factor and *STAT1*—a transcription regulator, were found to be activated in T helper cells, T cytotoxic cells, and B lymphocytes and inactivated in monocytes. The genes governed by the IRF7 and *STAT1* were mostly ISGs including *IFIT3, MX1*, and *ISG15* (Supplementary Figures 12, 13). Also, KLF4 was found to be in the list of top 4 upregulated transcription factors in T helper cells (z score 2.62), T cytotoxic cells (z score −2.72), and B lymphocytes (z score −3.42). However, KLF4 was not found involved in monocytes (Supplementary Figure 14). Further, the comparative analysis of other upstream regulators (enzyme, GPCR, growth factors, ion channels, kinases, ligand-dependent nucleic acid receptors, peptidases, phosphates, translation regulators, transmembrane receptors, transporters) revealed *DDX58, EIF2AK2, S100A8, S100A9, C3, ICAM1*, and *PARP9* genes to be downregulated and *IFIH1, SAMSN1, EIF4E*, and *TNFAIP3* genes not to be differentially expressed in monocytes. On the contrary, these genes were upregulated in T helper cells, T cytotoxic cells, and B lymphocytes. The genes involved in *EIF2AK2* and *DDX58* network were mostly ISGs (Supplementary Figures 15, 16).

**Figure 9:**
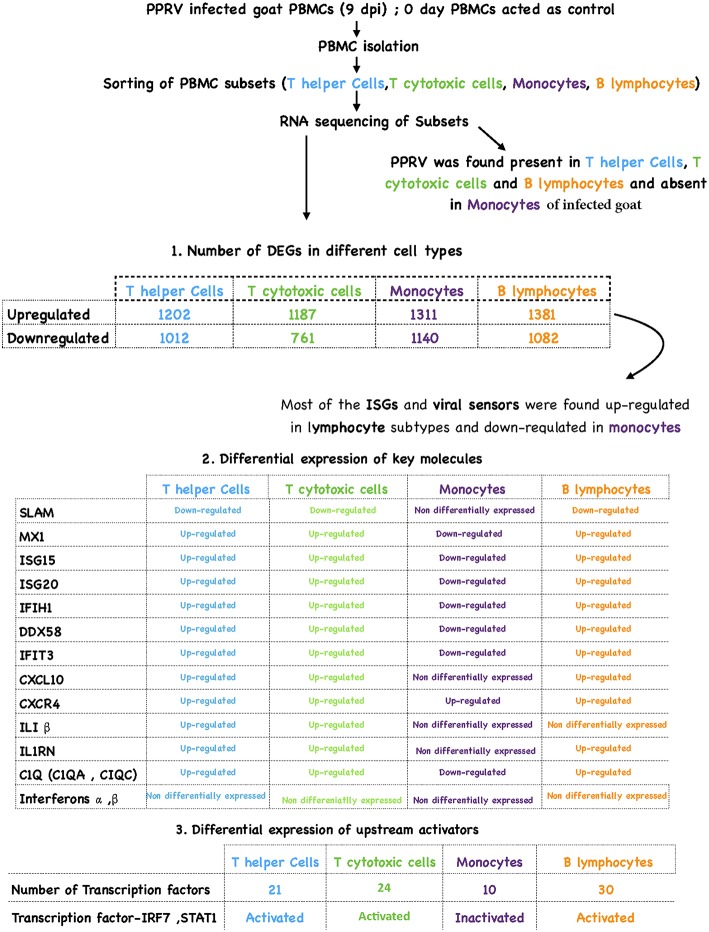
Overview of results.

The contrasting features between the monocytes and the lymphocytes are clearly given in the graphical abstract (Figure 9).

### DEGs Were Validated by qRT-PCR

**Figure 10:**
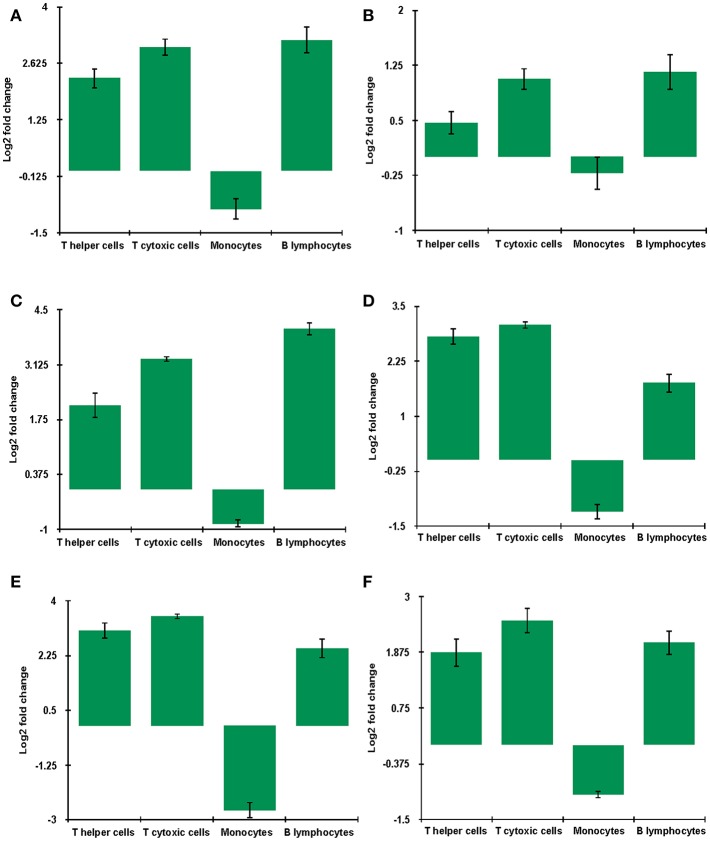
mRNA levels of genes **(A)** *DDX58* **(B)** *HERC5* **(C)** *IFIT3* **(D)** *IRF7* **(E)** *ISG15* **(F)** MX1 in unvaccinated-infected goats at 9 dpi were validated using quantitative qRT-PCR with GAPDH as a reference gene for normalization. Log2 fold change calculated by delta-delta Ct method with control as the calibrator is represented along with the standard error of the difference.

Key genes identified from RNA sequencing data—*DDX58, HERC5, IFIT3, IRF7, ISG15*, and *MX1* at 9 dpi were validated by qRT-PCR. The expression of all the validated genes was in concordance with RNA sequencing results (Figure 10).

## Discussion

Transcriptome analysis of subsets of PBMCs and analysis of cell-specific pathways facilitate in understanding the regulatory networks involved under PPRV infection. Virulent PPRV has been detected in PBMCs (4, 9, 33–36). No experimental evidence is available regarding the immune cell types that are infected with virulent PPRV. Further, due to the fact that each of the cell types has a program inherited for generating a distinct immune response, the analysis of each cell type under PPRV infection attains significance (17). We, therefore, conducted transcriptome analysis at 0 days (control) and 9 dpi (a time that corresponds to the peak of viremia) of each subset of PBMCs to uncover the genes involved in host response and identify distinct transcriptional alterations in T helper cells, T cytotoxic cells, monocytes, and B lymphocytes.

In our study, expression of N gene in PPRV was detected in infected goats at 9 dpi in lymphocytes (T helper cells, T cytotoxic cells, and B lymphocytes) and not in monocytes which is in concordance with the alignment of reads with PPRV genome. Moreover, maximum viral transcripts were found in T helper cells in goats. These results indicate that the PPRV virulent virus probably replicates in lymphocytes and not in monocytes. The closely related viruses, Canine distemper virus (CDV) and Measles virus (MV) are reported to infect and replicate in T cells and not in monocytes (37–39). SLAM, the established receptor for PPRV, was found to be downregulated in T helper, T cytotoxic, and B lymphocytes and was not found to be differentially expressed in monocytes of the PRRV infected goats. The absence of any viral transcript in monocytes is consistent with the lack of differential expression of SLAM. The downregulation of SLAM expression with increased viral replication at 9 dpi in lymphocytes, may be due to superinfection exclusion phenomenon as observed in many viruses of human and veterinary importance (40). Superinfection exclusion is a phenomenon in which a pre-existing or established viral infection prevents the entry of same virus or a closely related virus. In our earlier PPRV host-pathogen interaction studies, SLAM was found to be downregulated in PBMCs (9).

Global transcriptome of monocytes indicated profound deviation from the lymphocytes as evident from a large number of unique upregulated genes (851) and downregulated genes (605) under PPRV infection. Differential expression analysis of immune cell types in trivalent inactivated influenza vaccine-induced immune response also showed unique transcriptomic expression profiles as well as changing biological networks (17). Among the genes differentially expressed under PPRV infection, there was predominant dysregulation in ISGs across all subtypes. Most of the ISGs and viral sensors were found to be upregulated in lymphocyte subtypes and downregulated in monocytes.

DEGs that were found to be involved in viral process, viral life cycle, and viral genome replication were mostly ISGs and few viral sensors, chemokines, and interleukins in all the subsets. RIG1-like Receptor signaling pathway as predicted through IPA analysis was least active in monocytes. The upregulated viral sensors in lymphocytes, MDA5 *(IFIH1)*, and R1G-1 *(DDX58)* activate NFKB and IRF3/7 (41–43) and LGP2, inhibits paramyxovirus-induced activation of IFN genes (44). This indicates that a powerful sensing mechanism to induce effector antiviral molecules exists in PPRV infected lymphocytes, and not in monocytes. Further, ISGs—ISG15, Mx1, Mx2, RSAD2, IFIT3, and IFIT5 have a protective effect against various RNA viruses (8, 9, 45–57). The upregulation of ISGs in lymphocytes and downregulation in monocytes suggests a predominant role of lymphocytes and poor involvement of monocytes in anti-viral response against PPRV.

However, the positive regulators of ISGs (58), type I interferons *(IFN-*α*/*β*)* were not differentially expressed in lymphocytes or monocytes. The upstream regulator analysis in lymphocytes revealed activation of transcription factors, IRF-7, and STAT-1 that regulate most of the ISGs under PPRV infection. Thus, it expected that ISGs are transcriptionally induced more by IRF7 and STAT1 than by stimulation of interferons in PPRV infected lymphocytes. The presence of STAT1, which plays a vital role in interferon type I (IFN-α/β) and type II (IFN-γ) signaling (59) and the upregulation of ISGs substantiate for the activation of Interferon signaling pathway in B lymphocytes, T helper cells, and T cytotoxic cells under PPRV infection. On the contrary, inactivation of Interferon signaling pathway, IRF-7, and STAT-1 along with downregulation of ISGs was observed in monocytes.

IFN-α/β are mostly secreted by Plasmacytoid dendritic cells (pDCs) (60). Any kind of perturbance in their expression is appreciable in pDCs than in T or B cells. Type I interferons (IFN-α/β) were not differentially expressed in both lymphocytes or monocytes meaning that their expression is undeterred in B and T cells under PPRV infection. It is likely that PPRV is inhibiting the expression of IFN-α/β in pDCs and not in T or B cells. However, this warrants further studies.

Chemokine-*CXCL10* that was upregulated in the T helper cells, T cytotoxic cells, and B lymphocytes and not differentially expressed in monocytes have been reported to have chemoattractant (61). *IL-1*β that was found upregulated in lymphocytes and not differentially expressed in monocytes, is a potent inflammatory cytokine involved in the recruitment of immune and inflammatory cells into the site of infection and influences the development of adaptive immune responses (62). This suggests the role of lymphocytes in the recruitment of immune cells and induction of apoptosis in PPRV infected cells. Further, Complement factors- *C1QA, C1QBP*, and *C1QC* were upregulated in lymphocytes and downregulated in monocytes indicating strong virus opsonization by lymphocytes. C1q (*C1QA, C1QB, C1QC*) stimulates the hemagglutination and neutralization activity (63).

Our data suggest that important cell type-specific information is gained through transcriptome analysis of PBMCs subsets than from PBMCs as a whole. The presence of PPRV; downregulation of SLAM receptor; upregulation of viral sensors—MDA5 and DDX58; activation of upstream regulators—IRF7 and STAT1; activation of interferon signaling pathway and; upregulation of ISGs in lymphocytes with a contrast in monocytes indicated the predominant role of lymphocytes in generating the antiviral response against PPRV in goats.

## Data Availability

The datasets for this study were deposited in GEO (GSE132429).

## Ethics Statement

The study was carried out after obtaining permission from Indian Veterinary Research Institute Animal Ethics Committee (IVRI—IAEC) under the Committee for the Purpose of Control and Supervision of Experiments on Animals (CPCSEA), India. The protocols were approved vide letter no 387/CPSCEA.

## Author Contributions

RS, BPM, and RG conceived and designed the research. KR and DM performed the vaccine testing experiment. YS maintained the server for analysis. SW, ARS, DC and SS conducted the wet lab work. SW, RK, ARS, AP, VS, SK, and RG analyzed the data. SW, RG, RK, WM, APS, NH, AT, BS, and BM helped in manuscript drafting and editing. RS, BPM, and RG proofread the manuscript.

### Conflict of Interest Statement

The authors declare that the research was conducted in the absence of any commercial or financial relationships that could be construed as a potential conflict of interest. The reviewer NS and handling editor declared their shared affiliation.
