# Supplementary material for: Contrasting Gene Expression Profiles of Monocytes and Lymphocytes From Peste-Des-Petits-Ruminants Virus Infected Goats
Source: Front Immunol. 2019 Jul 5;10:1463. doi: 10.3389/fimmu.2019.01463 (PMC6624447; doi:10.3389/fimmu.2019.01463)
Supplement: Supplementary file 1 [file Data_Sheet_1.PDF]

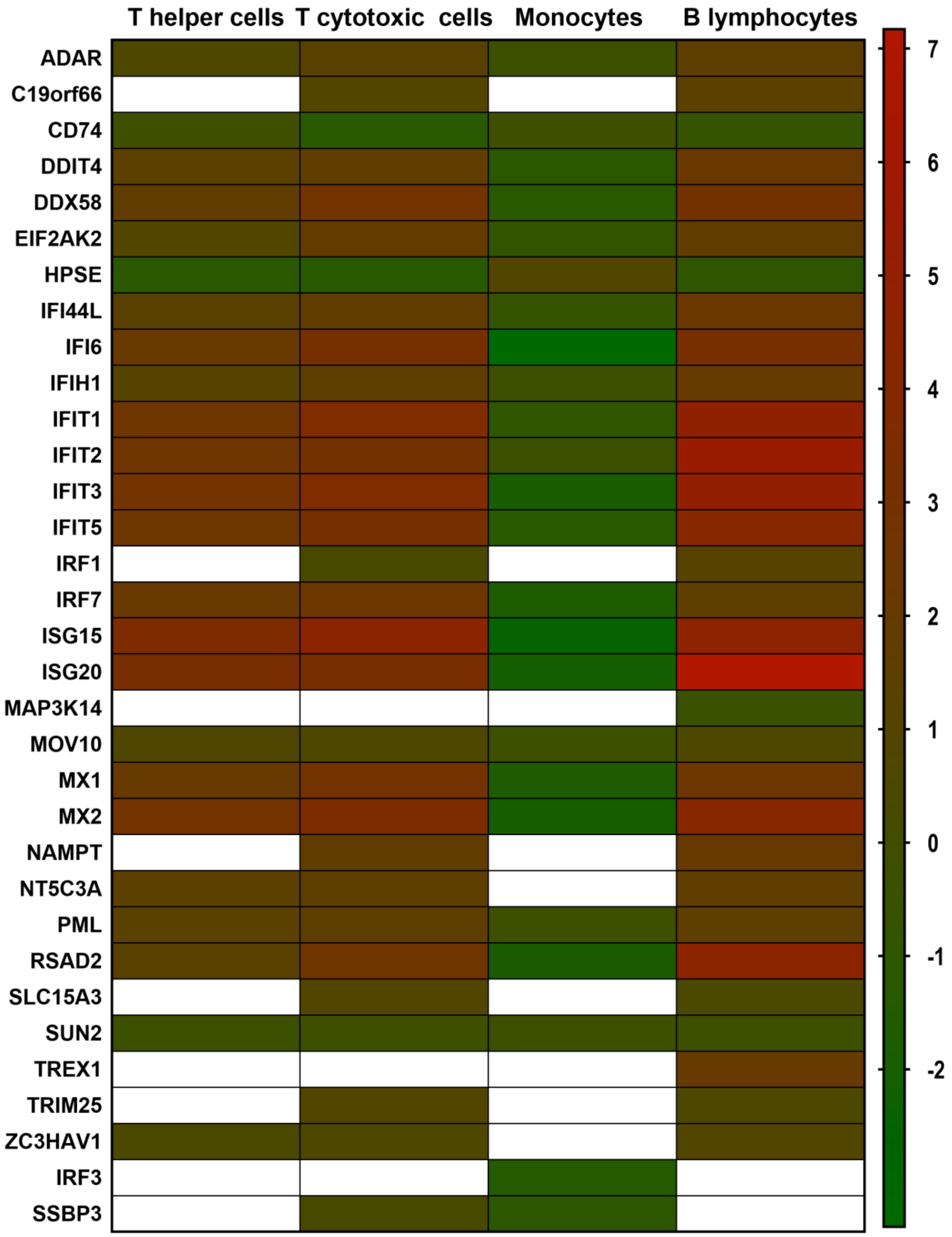

Supplementary Figure 1: Heat maps representing ISGs dysregulated in PBMC subsets of infected goats at 9 dpi.

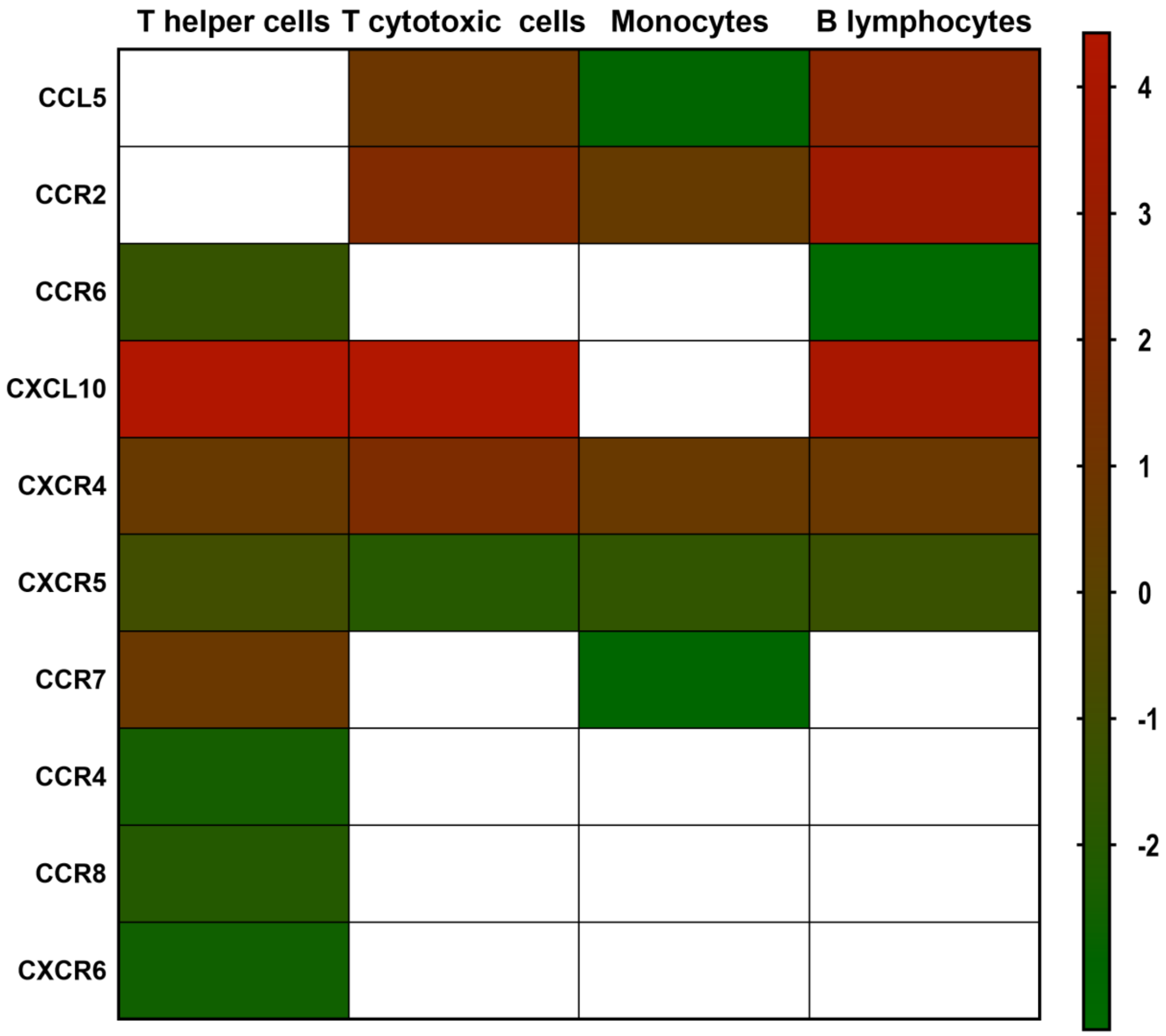

Supplementary Figure 2 (A)**:** Heat maps representing chemokines dysregulated in PBMC subsets of infected goats at 9 dpi.

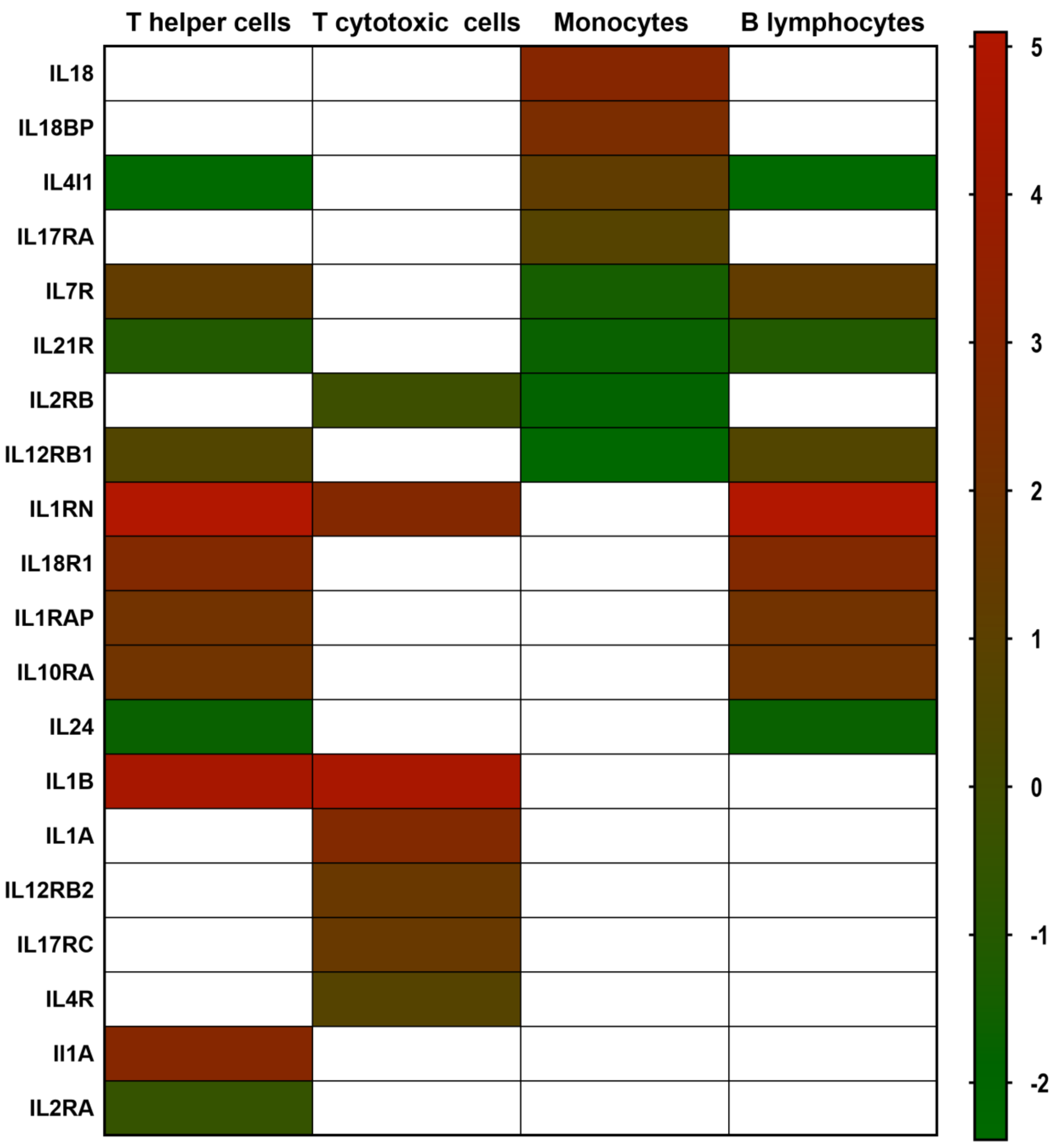

Supplementary Figure 2 (B)**:** Heat maps representing Interleukins dysregulated in PBMC subsets of infected goats at 9 dpi.

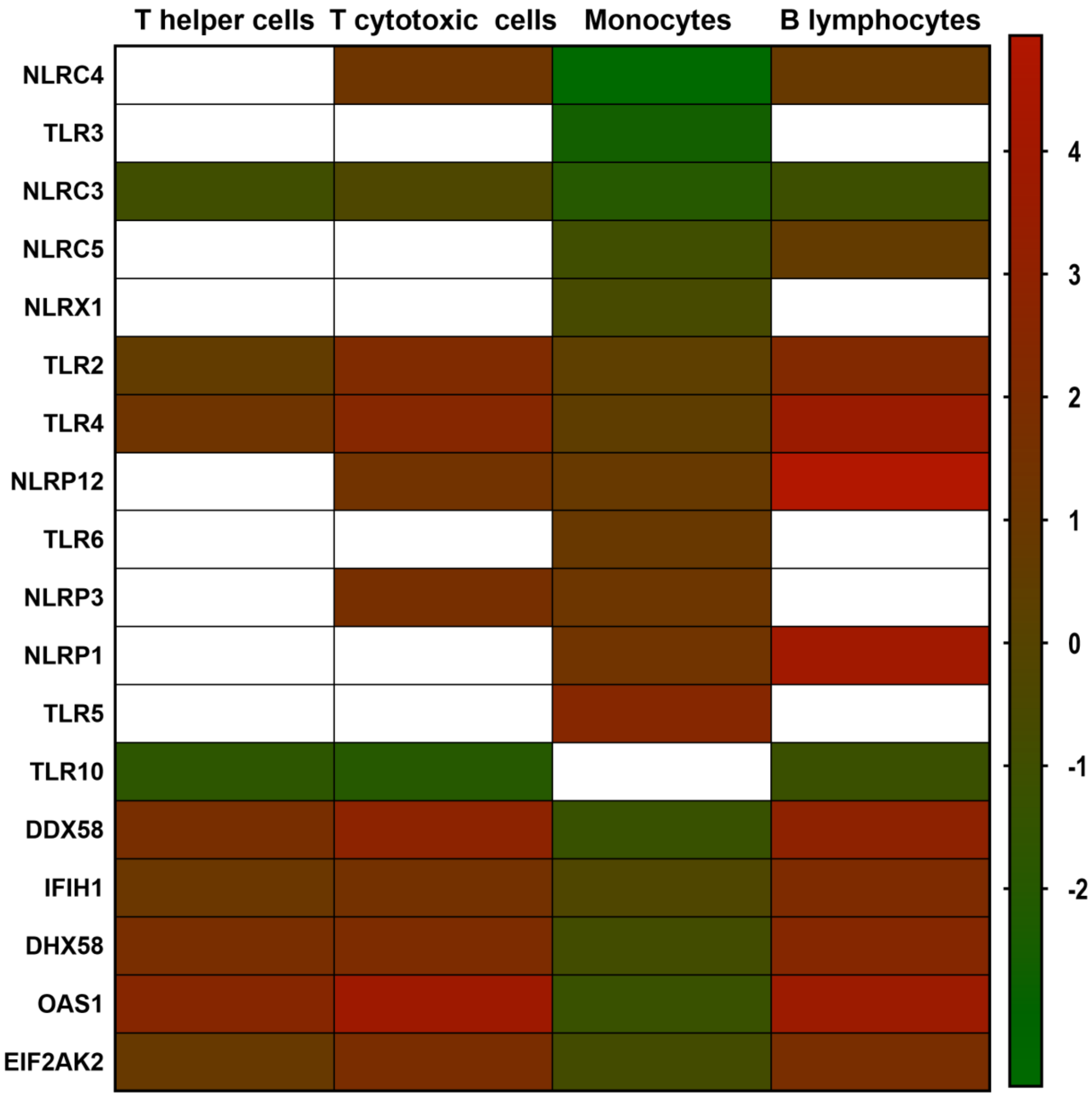

Supplementary Figure 3(A) **:** Heat maps representing viral sensors dysregulated in PBMC subsets of infected goats at 9 dpi.

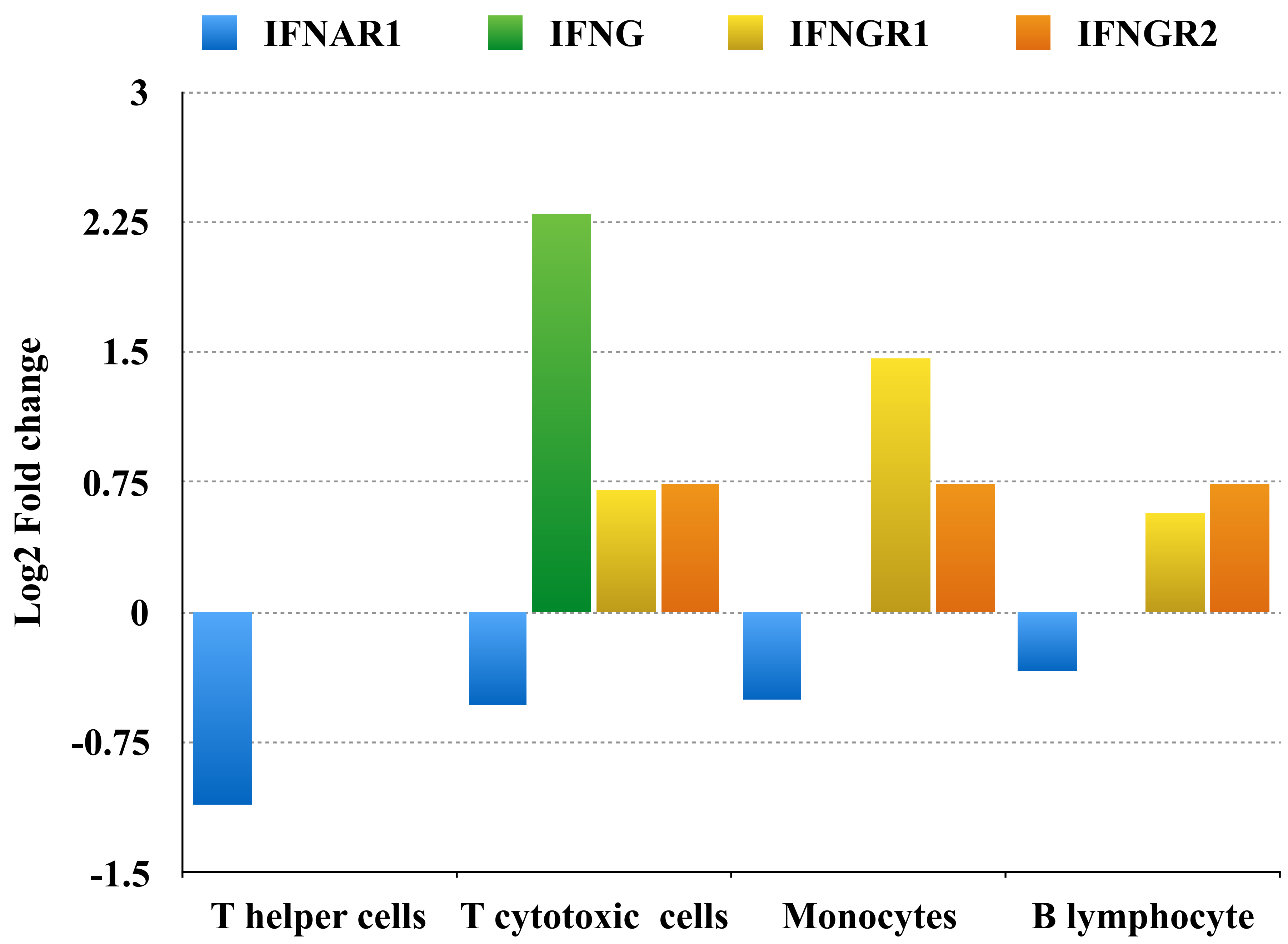

Supplementary Figure 3 (B): Heat maps representing interferons dysregulated in PBMC subsets of infected goats at 9 dpi.

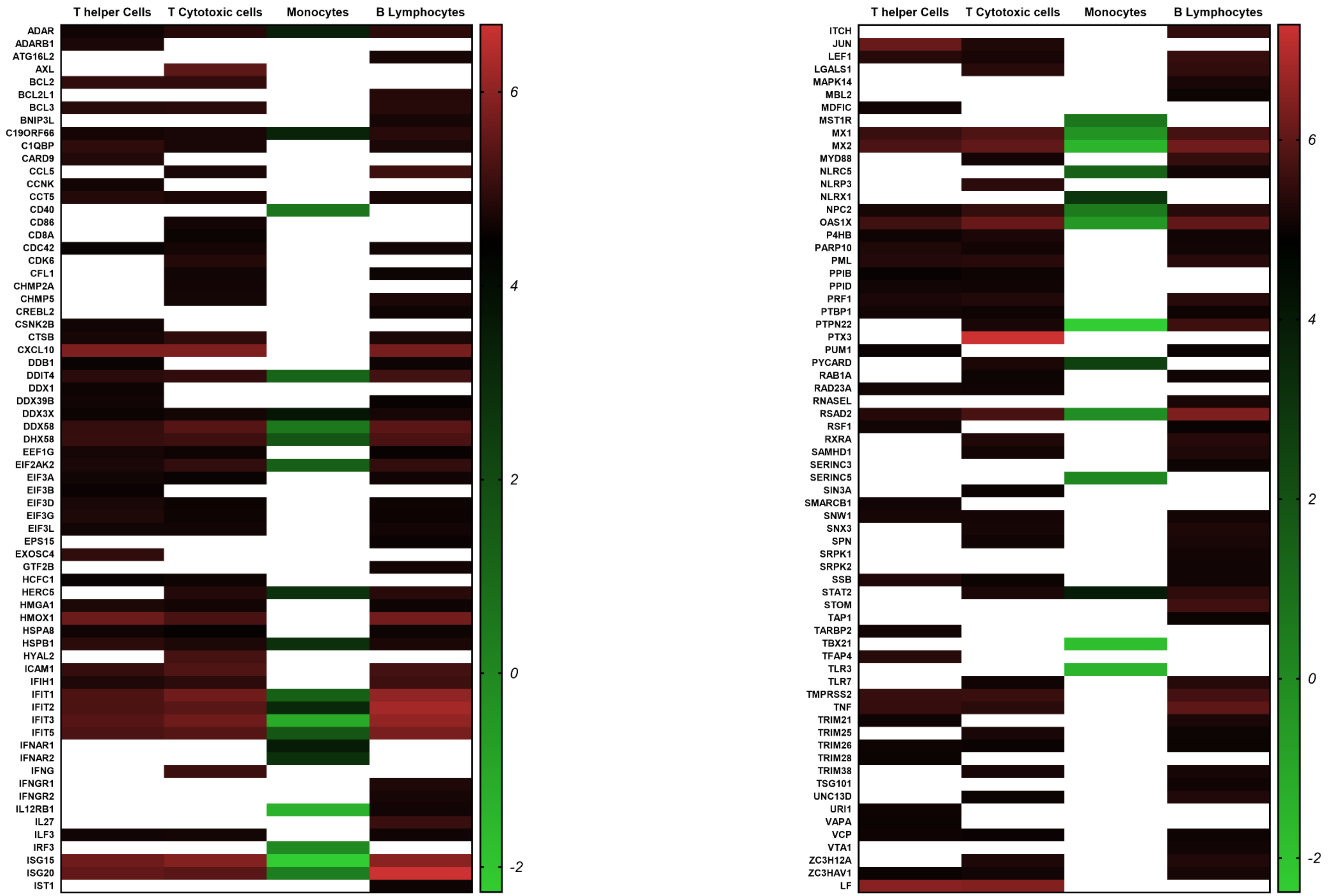

Supplementary Figure 4: Heat maps representing DEGs enriched in pathways of viral process, viral life cycle, viral genome replication etc. of infected goats at 9 dpi.

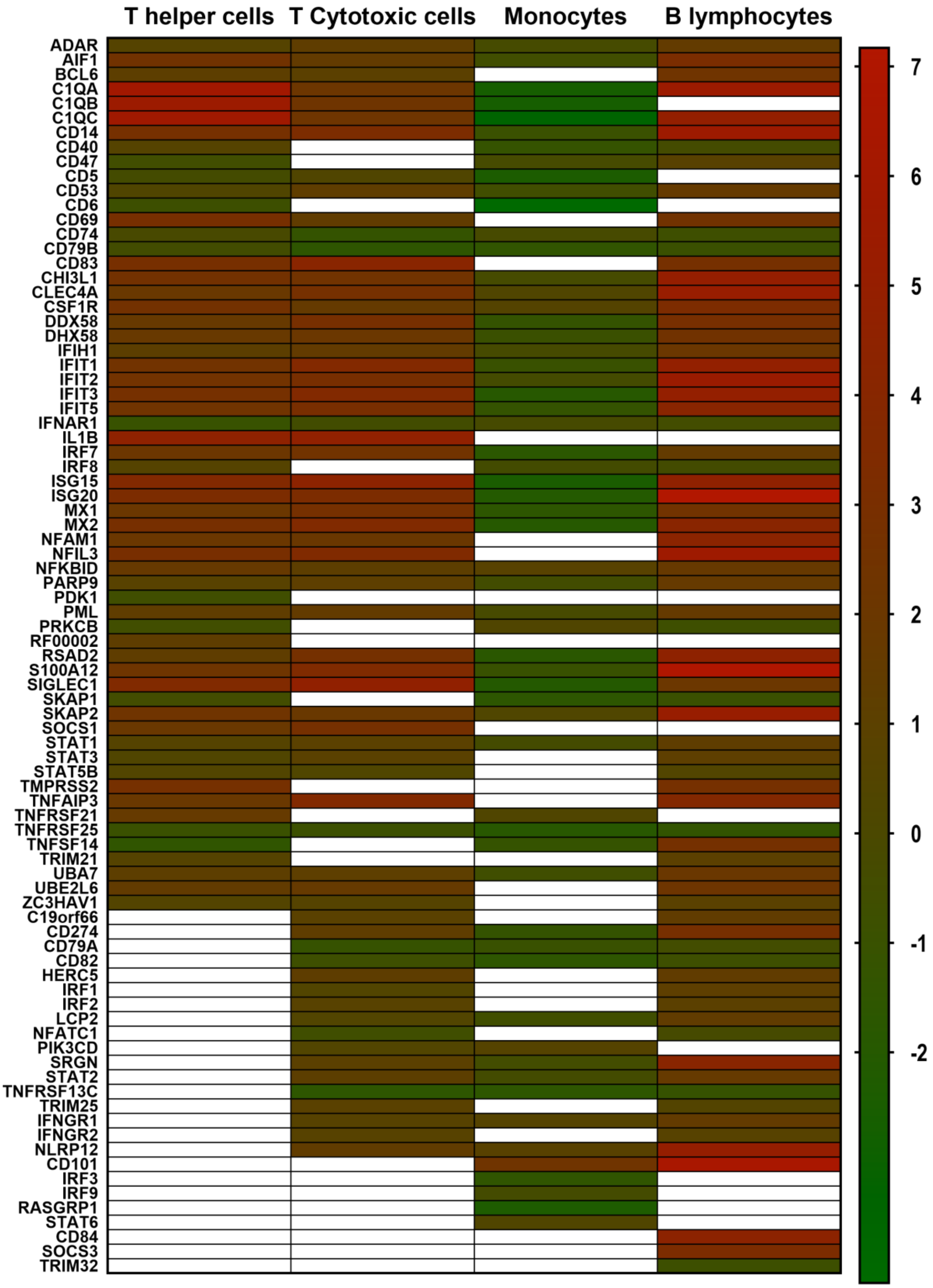

Supplementary Figure 5: Heat maps representing DEGs selected through knowledge based approach of infected goats at 9 dpi.

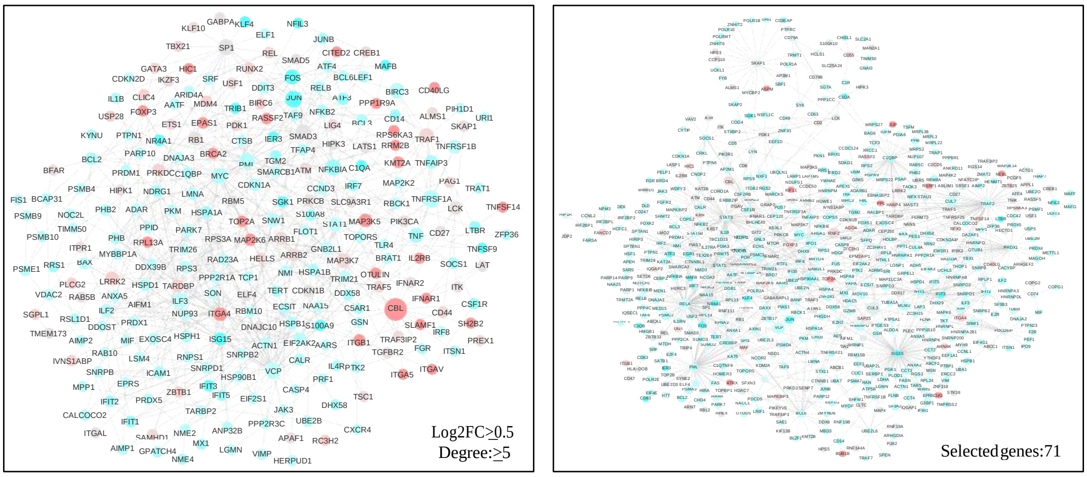

(A) (B)

Supplementary Figure 6: Protein-protein interaction networks in T helper cells of infected goats at 9 dpi. (A) Immune DEHC-DEHC network. (B) Selected genes and DEGs network. Turquoise blue colour of nodes indicates up regulation and carnation pink indicates down regulation of the genes with the gradient showing the extent of expression (log2 fold change). The diameter of the node represents the connectivity/degree of the node among the genes.

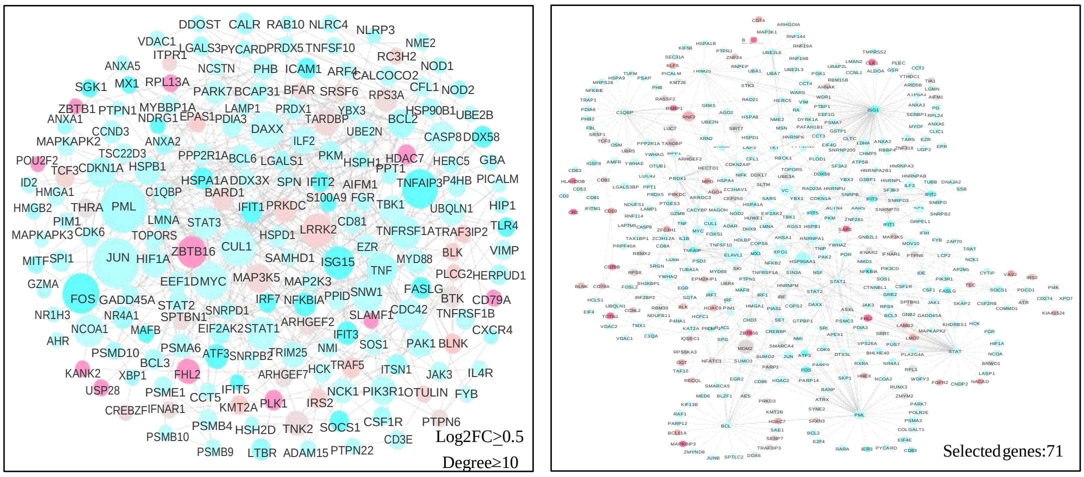

(A) (B)

Supplementary Figure 7: Protein-protein interaction networks in T cytotoxic cells of infected goats at 9 dpi. (A) Immune DEHC-DEHC network. (B) Selected genes and DEGs network. Turquoise blue colour of nodes indicates up regulation and carnation pink indicates down regulation of the genes with the gradient showing the extent of expression (log2 fold change). The diameter of the node represents the connectivity/degree of the node among the genes.

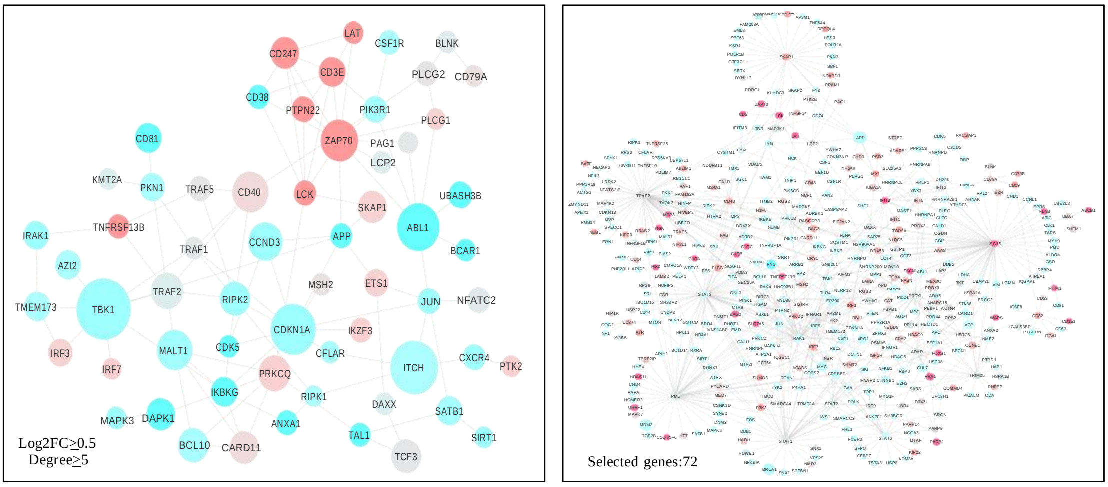

(A)

(B)

Supplementary Figure 8: Protein-protein interaction networks in monocytes of infected goats at 9 dpi. (A) Immune DEHC-DEHC network. (B) Selected genes and DEGs network. Turquoise blue colour of nodes indicates up regulation and carnation pink indicates down regulation of the genes with the gradient showing the extent of expression (log2 fold change). The diameter of the node represents the connectivity/degree of the node among the genes.

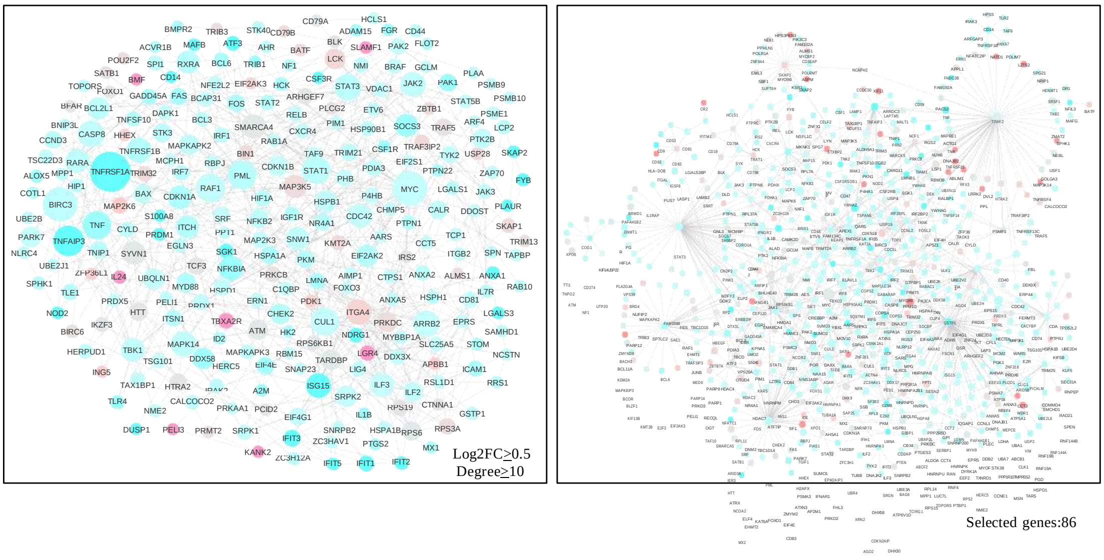

(A)

(B)

Supplementary Figure 9: Protein-protein interaction networks in B lymphocytes of infected goats at 9 dpi. (A) Immune DEHC-DEHC network. (B) Selected genes and DEGs network. Turquoise blue colour of nodes indicates up regulation and carnation pink indicates down regulation of the genes with the gradient showing the extent of expression (log2 fold change). The diameter of the node represents the connectivity/degree of the node among the genes.

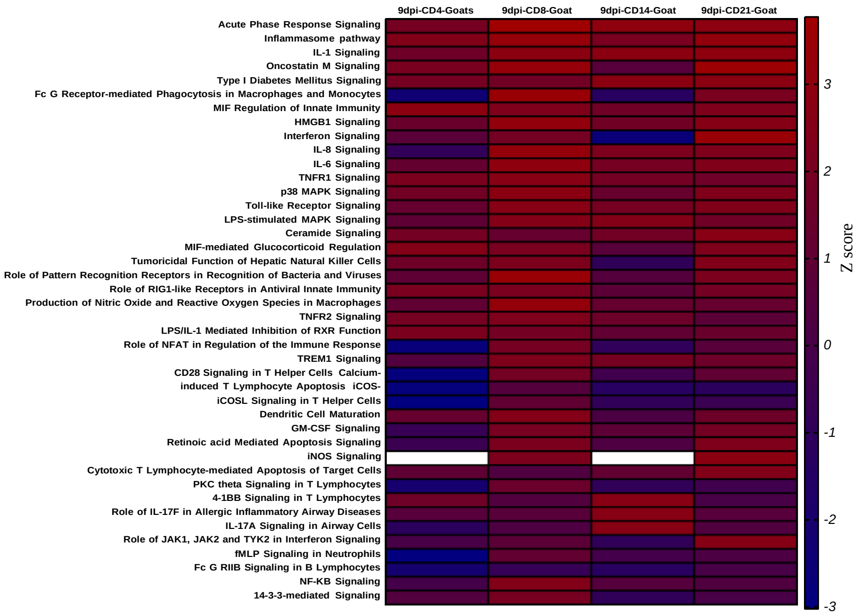

Supplementary Figure 10: Comparison analysis of canonical pathways activated / non-activated among T helper cells, T cytotoxic cells, monocytes, and B lymphocytes in infected goats at 9 dpi generated in Ingenuity pathway analysis tool. The Z score of the pathways > 2 indicates activation and < 2 inactivation.

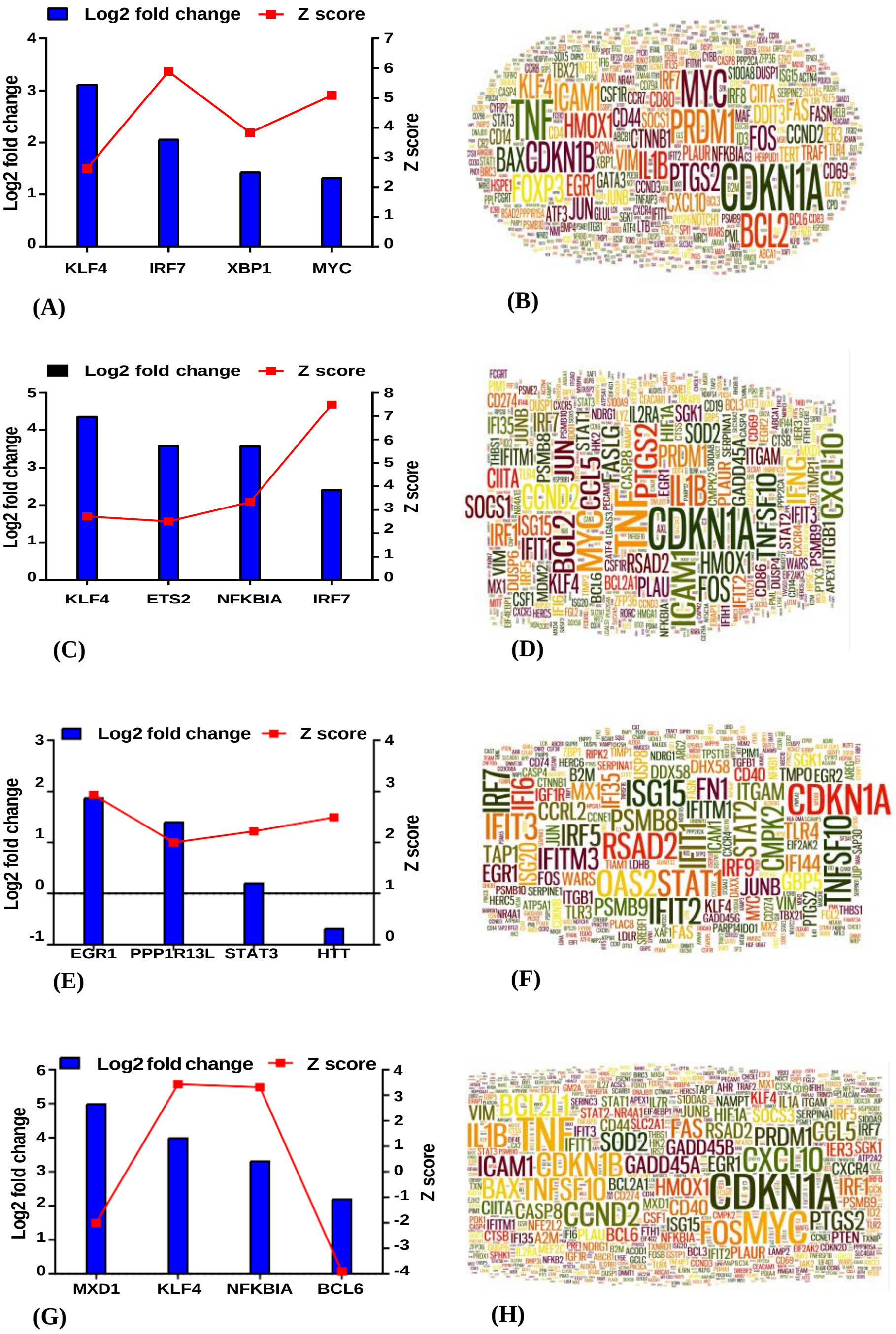

Supplementary Figure 11: Top four upregulated TFs governing the DEGs in (A) T helper cells, (C) T cytotoxic cells, (E) monocytes and (G) B lymphocytes of infected goats at 9 dpi were identified by upstream regulator prediction analysis of ingenuity pathway analysis tool. Height of the bar graphs in primary y axis indicates log2 fold change and the line graphs in secondary y axis shows Z score. The downstream target DEGs governed by all TFs in (B) T helper cells, (D) T cytotoxic cells, (F) monocytes and (H) B lymphocytes were represented in word clouds. The clouds give greater prominence to genes that were more frequent as targets of TFs.

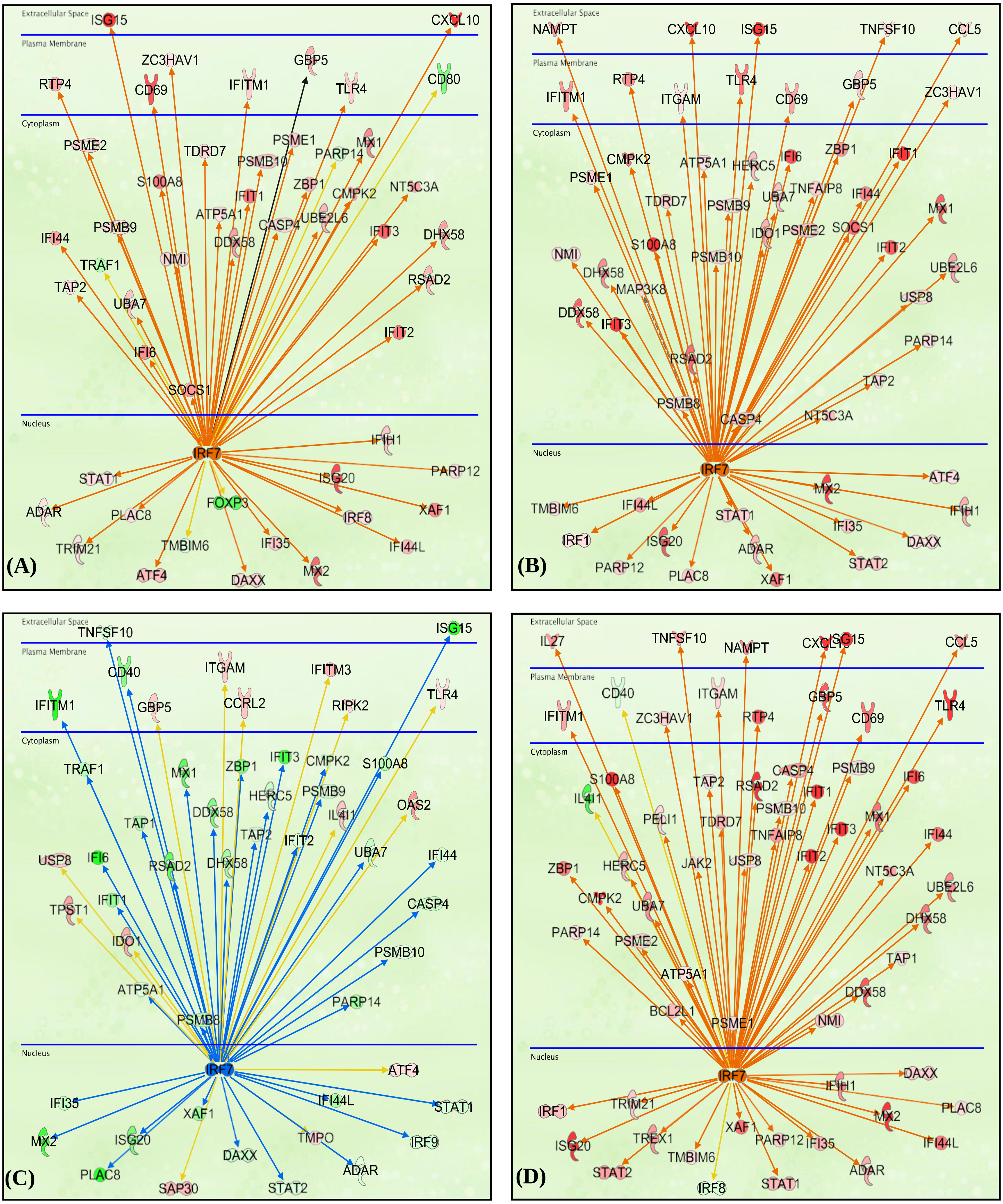

Supplementary Figure 12: IRF7 - upstream regulator governed downstream genes network generated by Ingenuity Pathway Analysis tool in (A) T helper cells, (B) T cytotoxic cells, (C) monocytes and (D) B lymphocytes of infected goats at 9 dpi. Genes that were upregulated are shown in red and downregulated in green. Symbol shape indicates gene function. Lines show predicted inhibition (blue) or activation (orange) of the downstream genes, per IPA.

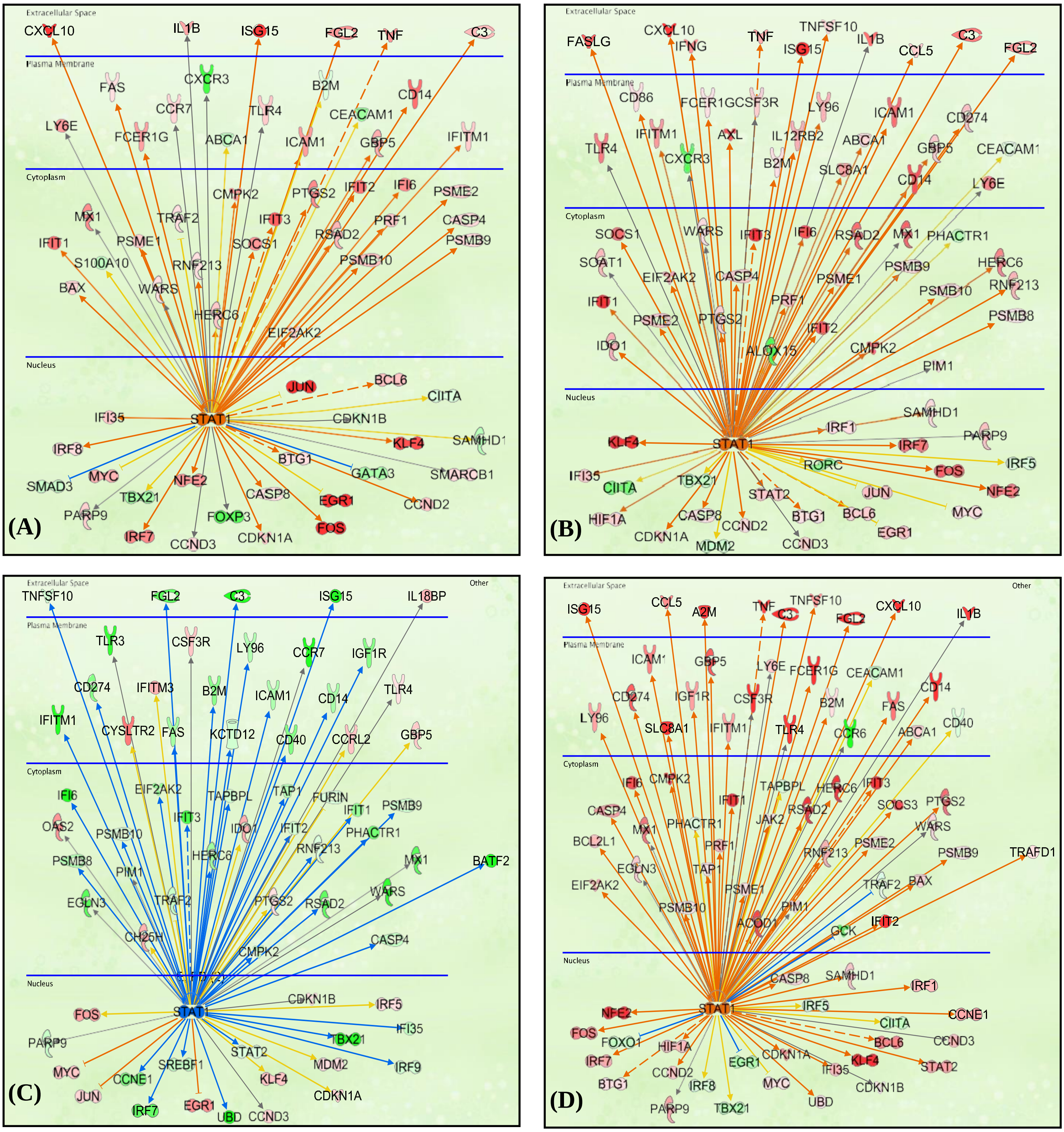

Supplementary Figure 13: STAT1 - upstream regulator governed downstream genes network generated by Ingenuity Pathway Analysis tool in (A) T helper cells, (B) T cytotoxic cells, (C) monocytes and (D) B lymphocytes of infected goats at 9 dpi. Genes that were upregulated are shown in red and downregulated in green. Symbol shape indicates gene function. Lines show predicted inhibition (blue) or activation (orange) of the downstream genes, per IPA

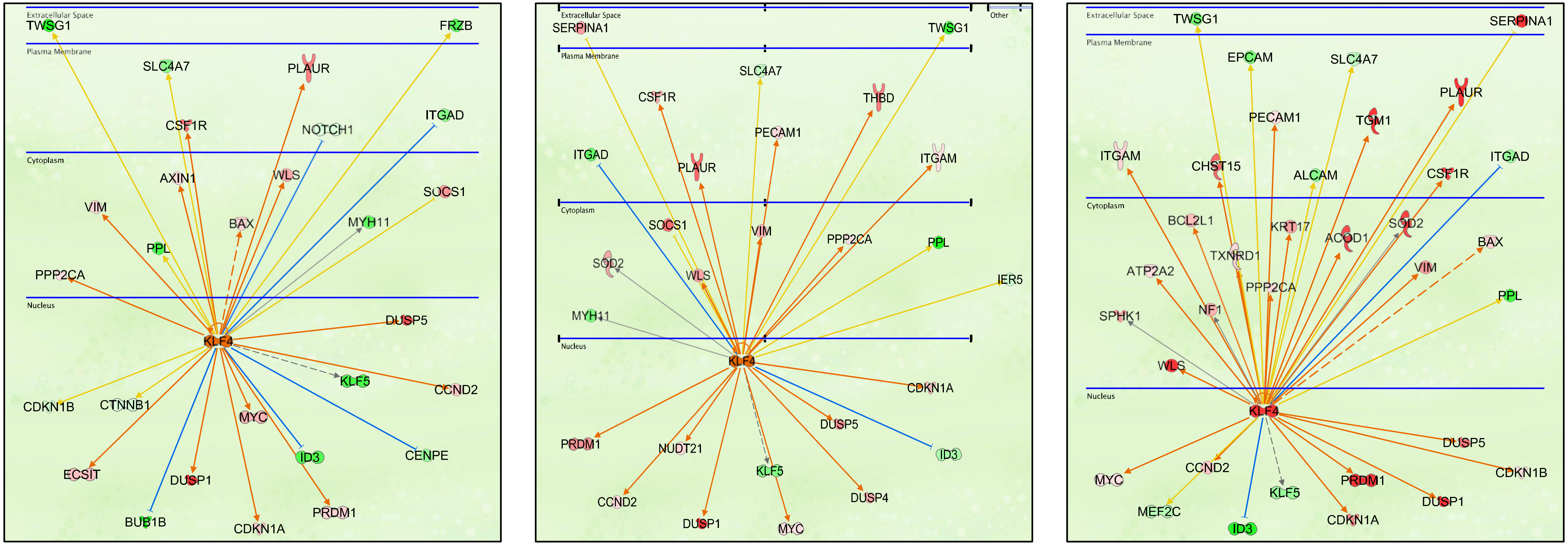

(A) (B) (C)

Supplementary Figure 14: KLF4-upstream regulator governed downstream genes network generated by Ingenuity Pathway Analysis tool in (A) T helper cells, (B) T cytotoxic cells and (C) B lymphocytes of infected goats at 9 dpi. Genes that were upregulated are shown in red and downregulated in green. Symbol shape indicates gene function. Lines show predicted inhibition (blue) or activation (orange) of the downstream genes, per IPA

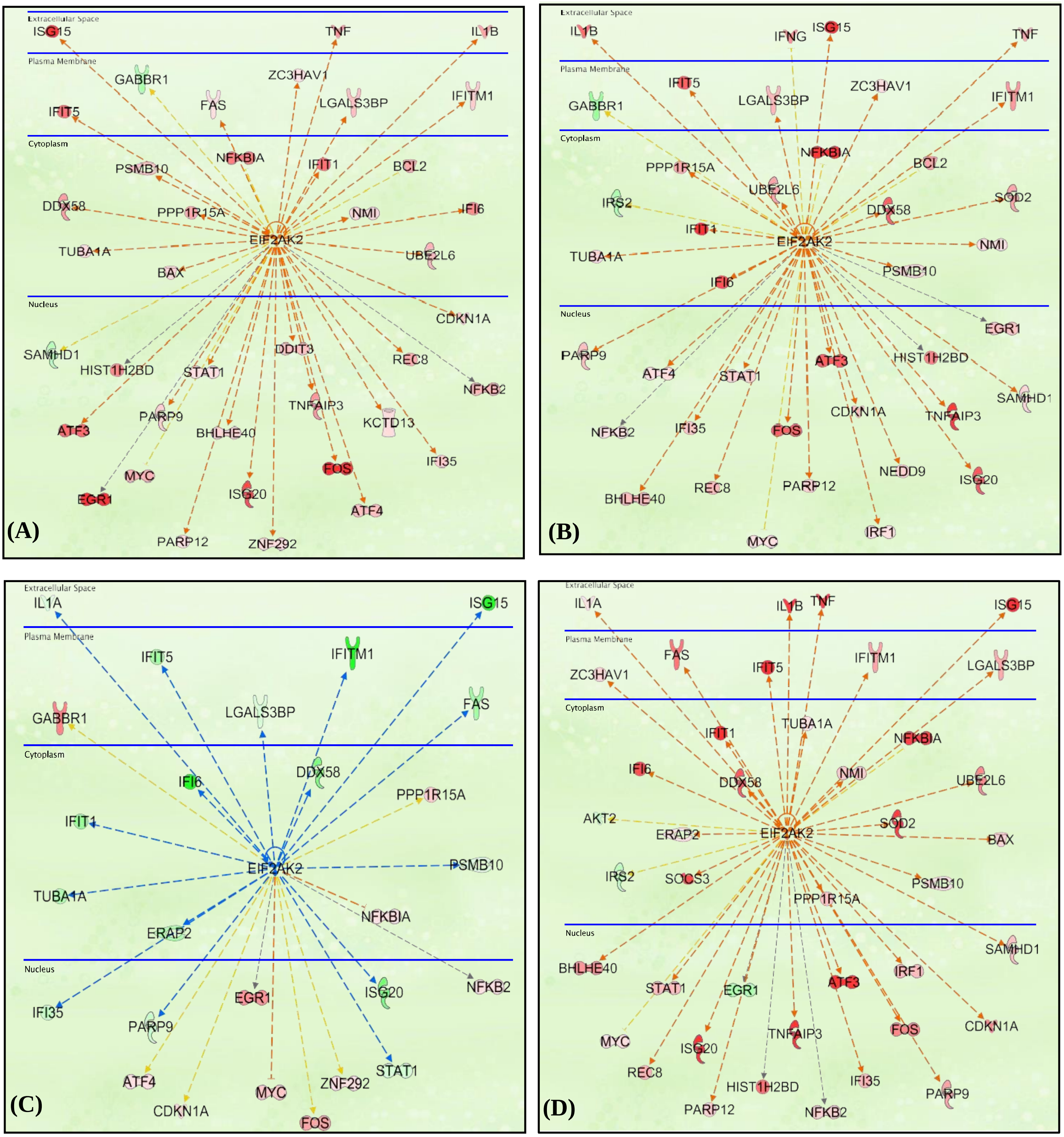

Supplementary Figure 15: EIF2AK2 - upstream regulator governed downstream genes network generated by Ingenuity Pathway Analysis tool in (A) T helper cells, (B) T cytotoxic cells, (C) monocytes and (D) B lymphocytes of infected goats at 9 dpi. Genes that were upregulated are shown in red and downregulated in green. Symbol shape indicates gene function. Lines show predicted inhibition (blue) or activation (orange) of the downstream genes, per IPA

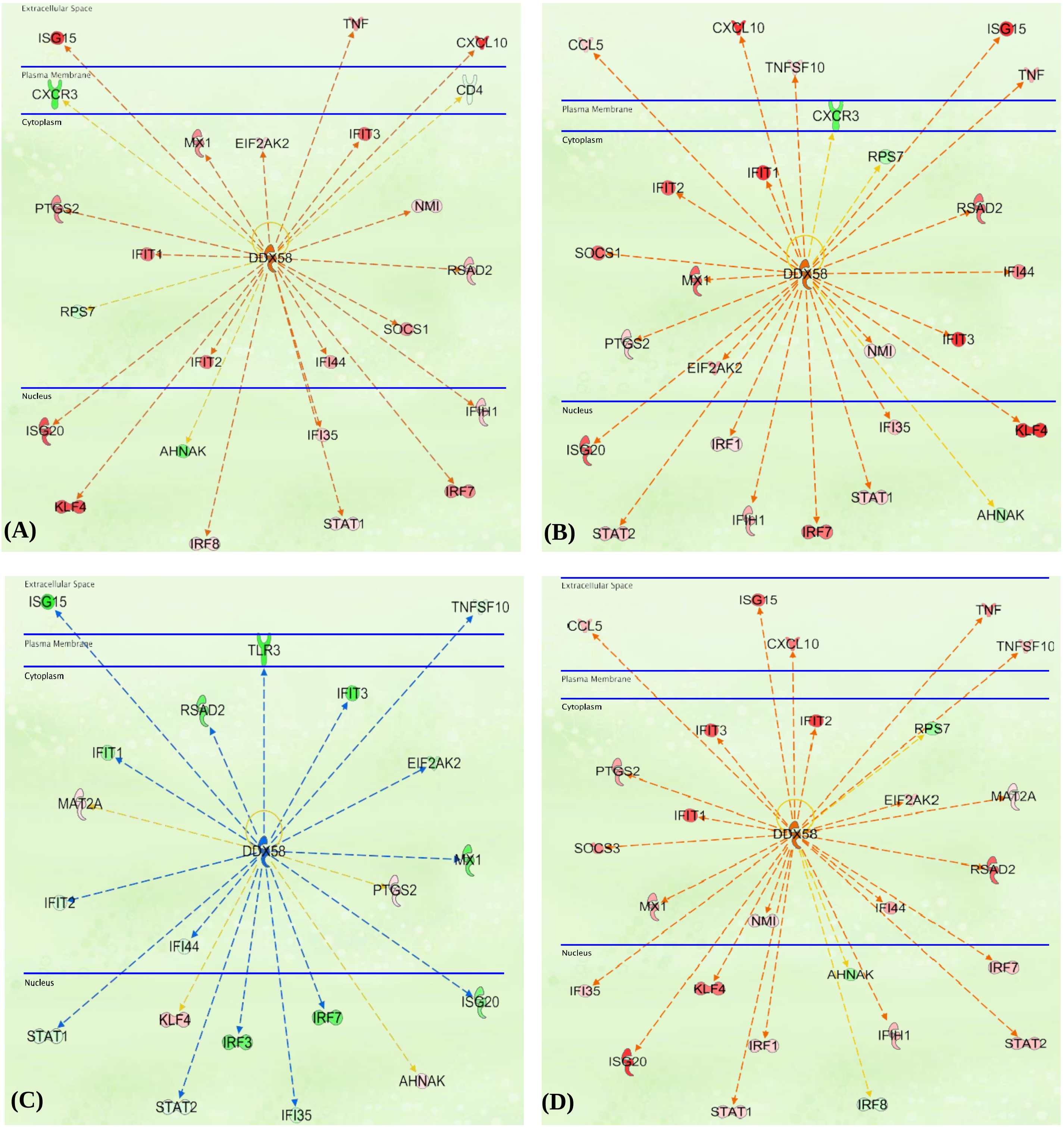

Supplementary Figure 16: DDX58 - upstream regulator governed downstream genes network generated by Ingenuity Pathway Analysis tool in (A) T helper cells, (B) T cytotoxic cells, (C) monocytes and (D) B lymphocytes of infected goats at 9 dpi. Genes that were upregulated are shown in red and downregulated in green. Symbol shape indicates gene function. Lines show predicted inhibition (blue) or activation (orange) of the downstream genes, per IPA

Supplementary Table 1: TaqMan probe ID of selected genes used in the study for validation by q-RT-PCR

Genes	TaqMan Assay ID
DDX58	Ch04684385_m1
IFIT3	AIAA1E0
IRF7	AI89L87
MX1	Oa04659431_m1
ISG15	AI70N2Z
HERC5	Ch04774461_m1
GAPDH	AIFAT31

Supplementary Table 2. List of the top 20 DEGs showing the most marked upregulation

T helper cells	Log2 fold change	T cytoxtic cells	Log2 fold change	Monocytes	Log2 fold change	B lymphocytes	Log2 fold change
C1QA	5.94	*IER3*	5.58	*FN1*	6.89	*S100A8*	7.45
C1QC	5.78	*LTF*	5.13	*ASIP*	5.96	*ISG20*	7.17
LTF	5.49	*SIGLEC1*	4.67	*AKR1E2*	4.88	*S100A12*	7.01
C1QB	5.43	*IL1B*	4.66	*F11R*	4.78	*C5AR2*	6.87
FOSB	5.31	*KLF4*	4.64	*ADAMTS13*	4.74	*PGLYRP1*	6.86
DYSF	4.92	*CXCL10*	4.43	*SULT1C4*	4.45	*PLAUR*	6.60
FOS	4.80	*ISG15*	4.41	*PNPLA7*	4.18	*CD101*	6.53
IL1B	4.53	*TRIB1*	4.38	*CD36*	4.10	*ETS2*	6.41
DUSP1	4.43	*ADGRE1*	4.34	*ADPRH*	4.00	*SDS*	6.36
CXCL10	4.36	*DRAM1*	4.20	*PIGR*	3.96	*P2RY13*	6.29
MAFB	4.24	*CD83*	4.09	*DNM1*	3.88	*LRG1*	6.25
IER3	4.21	*AXL*	4.06	*SNX33*	3.86	*SLC40A1*	6.22
CD3EAP	3.97	*CLEC4E*	4.06	*BST1*	3.81	*CRISPLD2*	6.17
NR4A1	3.93	*CD163*	4.05	*STON2*	3.77	*STEAP4*	6.16
KLF4	3.88	*ITGA9*	4.05	*PTGFRN*	3.74	*BCL2L15*	5.86
DUSP2	3.82	*S100A8*	4.01	*DLL1*	3.71	*MS4A8*	5.85
EGR1	3.74	*ETS2*	3.97	*GLT1D1*	3.71	*CEBPD*	5.80
JUN	3.63	*AQP9*	3.97	*TUBB6*	3.70	*SGK1*	5.77
RHOB	3.58	*DUSP1*	3.92	*CYBRD1*	3.57	*PSTPIP2*	5.73
ISG15	3.54	*NDRG1*	3.91	*MMP16*	3.56	*NFIL3*	5.64

Supplementary Table 3. List of the top 20 DEGs showing the most marked downregulation

T helper cells	Log2 fold change	T cytoxtic cells	Log2 fold change	Monocytes	Log2 fold change	B lymphocytes	Log2 fold change
CPNE6	-3.45	*NEBL*	-3.97	*ATP8*	-9.29	*DDIT4L*	-3.80
BUB1B	-3.42	*PTGS1*	-3.90	*TJP1*	-5.89	*SPDEF*	-3.63
SPDEF	-3.36	*ESYT3*	-3.59	*PPL*	-5.69	*CFAP58*	-3.52
FSTL1	-3.33	*PPM1J*	-3.54	*AMPD3*	-5.24	*CCR6*	-3.46
FCER2	-3.16	*FCER2*	-3.49	*FAM111B*	-4.94	*KIF12*	-3.34
ZBED6	-2.89	*SHISA8*	-3.30	*F2R*	-4.62	*HIST2H2AC*	-3.29
HFE	-2.87	*STX1A*	-3.21	*FAM241B*	-4.49	*FCER2*	-3.23
NEDD4	-2.81	*TRABD2B*	-3.10	*VLDLR*	-4.30	*C1orf115*	-3.18
EMP2	-2.80	*RF01877*	-3.04	*LCK*	-4.29	*DDR2*	-3.18
SEC14L5	-2.78	*INHBB*	-2.97	*MYH14*	-4.21	*KANK2*	-2.82
AQP3	-2.70	*WDR17*	-2.93	*OLFM1*	-4.15	*GMPR*	-2.77
ADAMTS7	-2.63	*IGFALS*	-2.89	*HIST1H2BD*	-4.14	*NCAN*	-2.77
ADAMTS18	-2.60	*FHL2*	-2.87	*CD6*	-4.11	*RAB37*	-2.74
CXCR6	-2.60	*GRIK4*	-2.77	*NID2*	-4.06	*OBSCN*	-2.71
JUP	-2.56	*KLHL3*	-2.75	*FMNL2*	-3.75	*TP53I11*	-2.69
PPM1J	-2.53	*DNAH7*	-2.75	*ALOX5*	-3.73	*LAMA5*	-2.68
CFAP58	-2.52	*CPT1B*	-2.70	*DST*	-3.70	*SCARA5*	-2.65
CD96	-2.50	*CFAP58*	-2.65	*CA2*	-3.68	*DENND2C*	-2.62
ACACB	-2.49	*STAC2*	-2.61	*RCCD1*	-3.65	*GALNT12*	-2.59
E2F8	-2.49	*GPR152*	-2.58	*GPR18*	-3.62	*TARSL2*	-2.55

Supplementary Table 4: Hubs in PPI networks of immune DEHC-DEHC genes in PBMC subsets

Cells	Infected goats
T helper cells	Up regulated: *VCP, ILF3, MYC, JUN, NFKBIA, PML, ISG15*
	Down regulated: *CBL, ITGA4, SP1, TRAF1, MAP3K7, ARRB2*
T cytotoxic cells	Up regulated: *PML, JUN, FOS, TNFAIP3, MYC, ISG15, STAT1, DAXX, CDKN1A, TNF, SNW1, TNFRSF1A, CASP8, CDC42*
	Down regulated: *LRRK2, MAP3K5, PRKDC*
Monocytes	Up regulated: *CDKNIA, TBK1, ABL1, IKBKG*

	Down regulated: *ZAP70, CD3E*
B lymphocytes	Up regulated: *TNFRSF1A, MYC, PML, RAF1, TNFAIP3, TNF, MAPK14, NFKBIA, SOCS3, STAT1, RAF1, STAT3, ISG15, ARRB2, TNFRSF1B, SOCS3*
	Down regulated: *ITGA4, MAP3K5, PRKDC, LGR4, SMARCA4*

Supplementary. Table 5: Hubs in PPI networks of selected genes and DEGs in PBMC subsets

Cells	Infected goats
T helper cells	Up regulated: *ISG15, PRDX1, PML. BCL6, C1QB, PRDX1, RELA, STAT3, STAT1 SOCS1*
	Down regulated: *SKAP1, CD55*
T cytotoxic cells	Up regulated: *ISG15, TRIM21, CIQB, STAT1, STAT3, PAL, BCL6, TNFAIP3, IFIT3, IFIT2, ELAVL1, SOCS1*
	Down regulated: *NFATC1*
Monocytes	Up regulated: *IRAK1, STAT3, PRKCB, STAT6, CREBBP, HSP90AA1*
	Down regulated: *ISG15, SKAP1, IRF3, STAT1, PML, TRAF2, IFIT3, CD40, TRIM25, PRDX1, CD82, CD40, CD274*
B lymphocytes	Up regulated: *ISG15, PML, BCL6, STAT3, TNFAIP3, IFIT3, STAT1*
	Down regulated: Nil
